# Myocardial Perfusion Simulation for Coronary Artery Disease: A Coupled Patient-Specific Multiscale Model

**DOI:** 10.1007/s10439-020-02681-z

**Published:** 2020-12-01

**Authors:** Lazaros Papamanolis, Hyun Jin Kim, Clara Jaquet, Matthew Sinclair, Michiel Schaap, Ibrahim Danad, Pepijn van Diemen, Paul Knaapen, Laurent Najman, Hugues Talbot, Charles A. Taylor, Irene Vignon-Clementel

**Affiliations:** 1 Inria, Paris, France; 2 HeartFlow Inc., Redwood City, USA; 3 LIGM, Université Gustave Eiffel, CNRS, ESIEE Paris, 77454 Marne-la-Valle, France; 4 Amsterdam UMC, Vrije Universiteit Amsterdam, Amsterdam, The Netherlands; 5 CentraleSupélec, Université Paris-Saclay, Inria, Gif-sur-Yvette, France; 6 Imperial College London, London, UK

**Keywords:** Heart, Hemodynamics, MBF (Myocardial blood flow), Coronary artery disease, PET perfusion map

## Abstract

Patient-specific models of blood flow are being used clinically to diagnose and plan treatment for coronary artery disease. A remaining challenge is bridging scales from flow in arteries to the micro-circulation supplying the myocardium. Previously proposed models are descriptive rather than predictive and have not been applied to human data. The goal here is to develop a multiscale patient-specific model enabling blood flow simulation from large coronary arteries to myocardial tissue. Patient vasculatures are segmented from coronary computed tomography angiography data and extended from the image-based model down to the arteriole level using a space-filling forest of synthetic trees. Blood flow is modeled by coupling a 1D model of the coronary arteries to a single-compartment Darcy myocardium model. Simulated results on five patients with non-obstructive coronary artery disease compare overall well to [$^{15}$O]$\text {H}_{{2}}$O PET exam data for both resting and hyperemic conditions. Results on a patient with severe obstructive disease link coronary artery narrowing with impaired myocardial blood flow, demonstrating the model’s ability to predict myocardial regions with perfusion deficit. This is the first report of a computational model for simulating blood flow from the epicardial coronary arteries to the left ventricle myocardium applied to and validated on human data.

**Electronic supplementary material:**

The online version of this article (10.1007/s10439-020-02681-z) contains supplementary material, which is available to authorized users.

## Introduction

Coronary artery disease (CAD), affecting millions of people each year, is the leading cause of death world-wide. Numerous cardiac exams are in clinical use for assessment of CAD, often relying on medical imaging to quantify anatomical and physiological measures prognostic for patient risk. Non-invasive testing modalities most widely utilized have demonstrated only modest diagnostic performance resulting in unnecessary hospital procedures^37^ costing billions of dollars annually. Some diagnostic modalities involve invasive protocols, putting patients at increased risk. With advances over the last few decades in medical imaging and functional modeling, patient-specific models (PSMs) have emerged as a non-invasive, cost-saving and integrative approach to assessing CAD.^42^

One of the remaining challenges in applying PSMs to quantify blood flow is to connect the disparate scales of cardiovascular physiology.^44^ The coronary arteries and the myocardium exhibit scale-specific properties and hence require different modeling approaches to sufficiently capture their behaviour at each scale.

Faced with the inherent challenges of modeling multiscale phenomena in the coronary circulation, many models focus either on the macro or micro vasculature. Taylor *et al*. describe a PSM to simulate blood flow inside large coronary arteries extracted from coronary Computed Tomography Angiography (cCTA) imaging data, to identify hemodynamically significant lesions via estimation of Fractional Flow Reserve (FFR).^42^ This method, named $\text {FFR}_{\text {CT}}$, relies on solving the 3D Navier-Stokes equations.^27^, ^42^$\text {FFR}_{\text {CT}}$ is cleared for clinical use by the U.S. Food and Drug Administration (FDA), has been extensively validated in multiple prospective clinical studies^15^, ^28^, ^32^, ^35^ and applied clinically in tens of thousands of patients to date.^36^ Other investigators have described alternate approaches for estimation of FFR with reduced order models of blood flow^18^, ^40^ or hybrid methods.^9^

However in this and other PSMs to model coronary blood flow, functional data is limited to the larger epicardial vessels due to the image resolution: the behaviour of the downstream coronary circulation is approximated by terminal vessel boundary conditions. Hence, although the effect of individual lesions in the epicardial coronary arteries on vessel blood flow can be modeled, the consequence of disease on blood flow to the myocardial tissue cannot be directly evaluated.

PSMs that seek to model the coronary microcirculation in more detail are still maturing. To handle the enormous number of small arteries, pre-arterioles, arterioles and capillaries in the modeling of whole heart coronary trees, most proposed methods leverage a porous-media model governed by Darcy’s law.^7^ Michler *et al.*^31^ have developed and tested a multi-compartment porous model in a porcine myocardium, using cryomicrotome data providing vessels down to the arteriole level. Limitations include the multicompartment computational cost and the difficulty in parameterizing the model with patient-specific data. Recently Alves *et al.*^2^ proposed a porous model applied to human data, which is able to simulate contrast agent transport and quantify perfusion. This model correlated well with perfusion MRI data, but was only applied to 2D slices of the myocardium.

A few multiscale models have been developed bridging functional analysis in large vessels with tissue perfusion. A major limitation is the anatomical gap between macro and micro scales. To extend the functional analysis, Smith *et al.*^41^ extrapolated a canine coronary anatomy from epicardial coronaries to small vessels by synthetic networks for the arterial and venous systems. 1D flow models in the latter were connected by 0D microcirculatory components: results exhibit realistic vessel pressure distribution, and inside the myocardium the spatial flow heterogeneity follows a fractal pattern as previously described.^3^ Hyde *et al.*^21^ used animal cryomicrotome data to connect epicardial arteries and microvessels, with a one-way coupling between a 1D coronary model and a multi-compartment porous model: inclusion of vascular data significantly improves the continuum perfusion results in comparison to a more simplistically parameterized model. Conversely, knowledge of perfusion^16^ and coronary flow repartition^9^, ^33^ may improve coronary model boundary conditions. Finally, Lee *et al.*^29^ proposed a two-way coupling between a 1D coronary model and a single compartment poromechanical model, applied to a porcine geometry extracted from cryomicrotome. This model reproduces layer-dependent perfusion pattern during the whole heart cycle. This framework also includes contrast agent transport modeling and was used to simulate perfusion images, demonstrating a perfusion deficit in the neighbouring region of a simulated stenosis.

So far all models connecting coronary blood flow to myocardial perfusion have been developed on animal data and are descriptive rather than predictive. These models demonstrate the expected characteristics of spatial flow distribution inside the myocardium, but have not been compared with ground-truth data. Furthermore, when relying on destructive image acquisition such as cryomicrotome, these methods are not suited for clinical application.

The aim of this work is to develop a multiscale patient-specific model on human data, enabling blood flow simulation from the large coronary arteries to myocardial tissue. The model is adapted to the patient’s coronary arterial network, which is extended with a synthetic vasculature.^23^ The computational model couples a 1D flow model in coronaries with a single compartment porous model for the myocardium. We applied this method to 6 patients with suspected CAD who underwent cCTA and [$^{15}$O]$\text {H}_{{2}}$O Positron Emission Tomography (PET)^13^ prior to invasive coronary angiography, which demonstrated non-obstructive CAD in 5 patients and obstructive CAD in 1 patient. We analyzed hemodynamic results both along the coronary vasculature and inside the myocardium with respect to the literature, and compared simulated perfusion with [$^{15}$O]$\text {H}_{{2}}$O PET perfusion data.

## Materials and Methods

Regarding ethics, this *post hoc* substudy comprises the 6 patients mentioned above, from the PACIFIC trial (NCT01521468). The study complied with the Declaration of Helsinki, the study protocol was approved by the VUmc Medical Ethics Review Committee, and all patients provided written informed consent.

This section further consists of five parts: generation of the coronary arterial network from the aorta down to the arteriole level, the one-dimensional blood flow model, the myocardium perfusion model, the coupling of the one-dimensional blood flow model and the myocardium model, and the definition of post-processed quantities.

**Figure 1:**
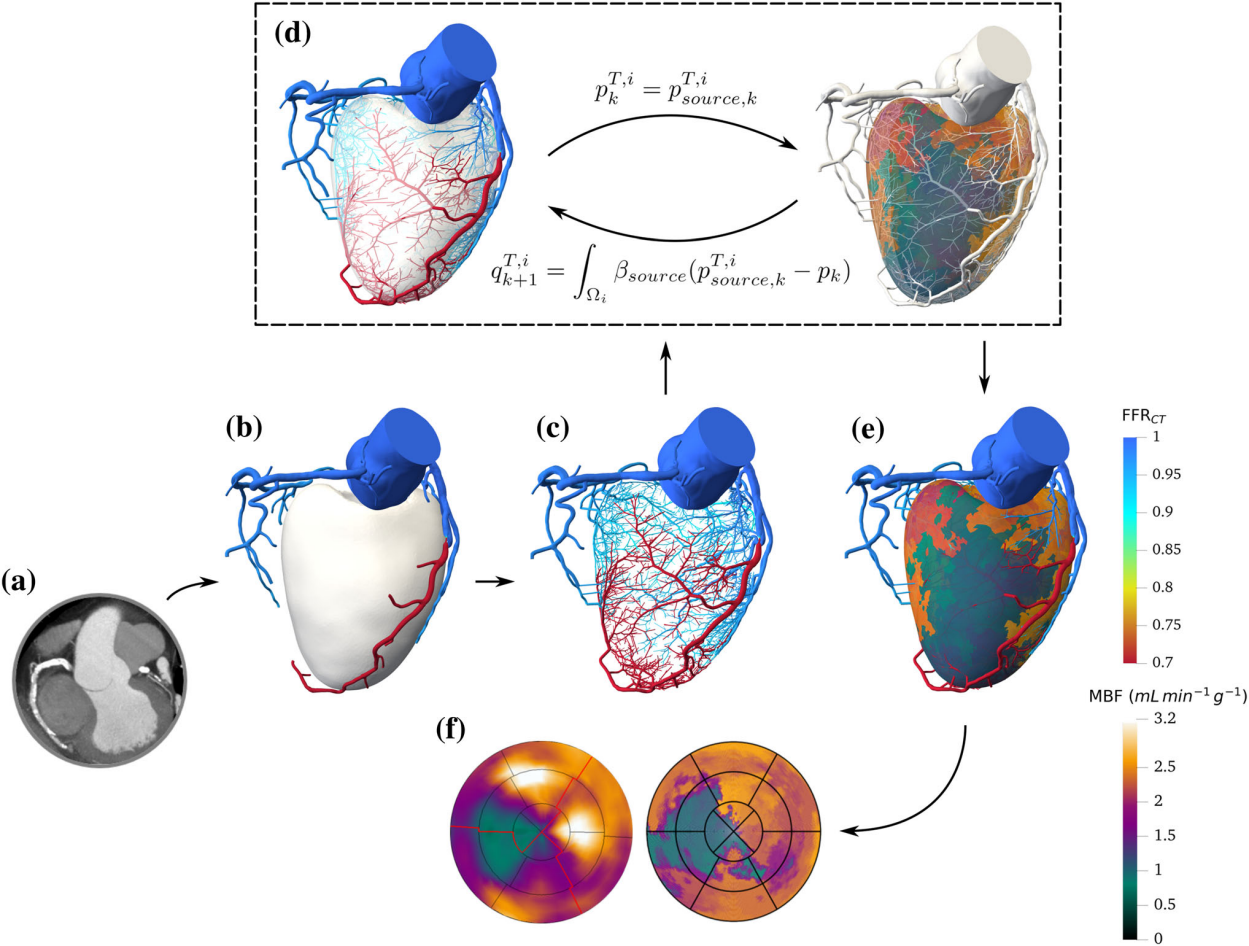
Illustration of the pipeline for a patient with disease in the left anterior descending coronary artery. (a) CT imaging data. (b) Segmented geometry and $\text {FFR}_{\text {CT}}$ analysis. (c) $\text {FFR}_{\text {CT}}$ results in segmented and synthetic vasculature. (d) Illustration of the coupling loop, demonstrating quantities exchanged between the coronary model (left) and the myocardium model (right) at coupling iteration *k*. (e) Hyperemic MBF for coupled model. (f) Comparison of simulated (right) and [$^{15}$O]$\text {H}_{{2}}$O PET (left) hyperemic perfusion maps.

In modeling the coronary arterial circulation, the aorta, epicardial coronary arteries and left ventricle myocardium are first segmented from patient cCTA image data using custom methods (HeartFlow Inc., US). The coronary trees are then extended from the image-based model down to the arteriole level using a space-filling synthetic forest of arterial trees. The downstream coronary circulation is either taken into account by a terminal resistance, when the coronary model is used stand-alone, or by a myocardium model. The latter is thus described along with its coupling with the coronary model. In both models, blood is considered as an incompressible Newtonian fluid. In this paper we only compute steady-state hemodynamic quantities, seeking to model only mean flow and pressure under resting and hyperemic conditions to study myocardial perfusion. Finally, relevant quantities are derived from the simulations for comparison with the literature and [$^{15}$O]$\text {H}_{{2}}$O PET perfusion data. The complete modeling pipeline is summarized in Fig. 1. Imaging protocols for cCTA and [$^{15}$O]$\text {H}_{{2}}$O PET are provided in Electronic Supplementary Material (ESM) section 1.

### Vascular Network Generation

The vascular networks are patient-specific hybrid vasculatures composed of (1) the aorta and epicardial coronary vessels segmented from cCTA and (2) synthetic trees of the downstream arteries. The synthetic coronary trees are generated as described in Jaquet *et al*.^23^ Briefly, synthetic tree roots are defined at segmented coronary outlets and additionally along the sides of the segmented vessels according to the patient’s branching pattern. These additional roots are added to represent the smaller branches expected to be missed in the segmentation due to the limited cCTA spatial resolution. Only synthetic tree roots close to the left ventricular myocardium are identified for vascular tree generation. A target flow to each tree root is estimated based on the root diameter and the total baseline flow defined later in Eq. 2. The synthetic tree is generated by minimizing the total generated vascular volume, and constrained by patient-specific priors which are the segmented vessels and left ventricular myocardium. The competitive growth between multiple synthetic trees is driven by tree target flows. Tree generation terminates once a certain number of terminal segments is reached.

The resulting hybrid vasculature is composed of two bifurcating trees, the left and right (RCA) coronary arteries branching of the aorta. The left side bifurcates into the left anterior descending (LAD) and left circumflex (LCX) arteries. Each vasculature is characterized by the number of terminal segments $n_{\text {term}}$. Each terminal segment outlet *i*, $i=1 \ldots n_{\text {term}}$, is identified in the manuscript with superscript $\text {T,i}$.

Due to initialization or geometrical constraints, some synthetic trees do not extend properly during the vascular growth computation. They do not reach their target flow and generate few terminal segments, typically with large diameters. To limit discrepancy between terminal segments and to improve scale consistency of the synthetic network, we remove those synthetic trees arising from the segmented vessels which reach less than 20% of their target flow.

### Coronary Model

#### Model Description

In the vascular network defined above, blood flow is modeled with the one dimensional approximation of the Navier–Stokes equations.^17^ Combined with the mass balance equation, the system of equations describing steady blood flow along the centerline axis of each vessel is:

1a $$\begin{aligned} \frac{\partial Q}{\partial z} = 0 \end{aligned}$$

1b $$\begin{aligned} \frac{\partial }{\partial z} \left( \alpha \frac{Q^{2}}{S}\right) + \frac{S}{\rho } \frac{\partial p}{\partial z} + 8 \pi \nu \frac{Q}{S} = 0 \end{aligned}$$

with *z* the coordinate of the centerline axis, *Q* and *p* the flow rate and pressure respectively, *S* the cross-sectional area of the vessel, $\alpha$ a geometry-related parameter, $\rho$ the blood density and $\nu$ the kinematic viscosity. Flow rate and pressure are respectively reported in $\text {mL}\,\text {min}^{-1}$ and $\text {mmHg}$. Blood density is set to ${1.06}\,\text {g}\,\text {cm}^{-3}$ and dynamic viscosity to ${0.053}\,\text {g}\,\text {cm}^{-1}\,\text {s}^{-1}$. The constant $\alpha = 4/3$ is obtained for a parabolic velocity profile. For area-increasing regions, $\alpha$ values are adjusted to prevent full pressure recovery downstream (Table 1). The values are empirically derived to minimize differences between 1D and 3D CFD solutions.

At each bifurcation of the vascular network, conservation of mass and continuity of pressure provide a relationship between vessel unknowns, completing the system of equations. In contrast to the segmented arteries which have variable vessel areas *S*, for synthetic vessels the system is practically reduced to Poiseuille law as *S* remains constant along each segment.

At the left and right coronary trees inlet, the average aortic pressure over a cardiac cycle is set to $P_{\text {AO}} = {93}\,\text {mmHg}$. The same aortic pressure is maintained at hyperemia, as FFR remains relatively constant with variations in aortic pressure values and heart rate.^14^,^24^

At terminal segments of the network, flow rate boundary conditions are imposed. Given these boundary conditions, flow rate solutions are first obtained for each segment up to the vasculature root. Pressure solutions can then be computed from the root down to the terminal segments. When solving the 1D equations, the flow rate boundary conditions and the synthetic tree geometry are updated iteratively to reflect rest and hyperemic conditions as described in the next section.

Note that segmented coronary arteries are discretized based on their centerline: 1D meshes consist of nodes and elements (connections between consecutively connected nodes). The spacing between two nodes is ${0.01}\,\text {cm}$. For the synthetic part of the vasculature, as the model is reduced to 0D, no spatial discretization is needed. The spatial location of each synthetic segment is tracked using starting and ending points. This framework has been implemented in an in-house code.

**Table 1:** Multiplication factor to the default $\alpha$ value of 4/3 for area-increasing regions under resting and hyperemic conditions.

Segmented vessel node	Multiplication factor value
Resting conditions	
In bifurcating region	0.29
Outside bifurcating region	0.34
Hyperemic conditions	
In bifurcating region	0.72
Outside bifurcating region	0.36

#### Parameterization for Rest and Hyperemic Conditions

*Resting Conditions* The total myocardial baseline flow $Q_{\text {rest}, \text {LV}}^{\text {tot}}$ is estimated from the segmented left ventricular myocardial volume $V_{\text {LV}}$, following the relationship defined by Choi *et al*.^8^:

2 $$\begin{aligned} Q_{\text {rest,LV}}^{\text {tot}} = \zeta \times {V_{\text {LV}}}^{\gamma } \end{aligned}$$

with $\gamma$ set to 0.75.^8^ The coefficient $\zeta$ is assigned a value of ${3.41}\,{\text {mL}^{0.25}\,\text {min}^{-1}}$. This value was determined empirically using a left ventricular myocardial mass distribution of patients enrolled in previously completed clinical studies^28^, ^34^ by fitting to a normal distribution of myocardial blood flow with a median of ${1}\,{\text {mL}\,\text {min}^{-1}\text {g}^{-1}}$.

The flow at each terminal segment that is perfusing the left ventricle is initialized based on its radius:

3 $$\begin{aligned} q^{\text {T,i}}_{\text {rest}} = \frac{(r^{\text {T,i}})^{2.7}}{\sum \nolimits _{i=1}^{n_{\text {term}}} (r^{\text {T,i}})^{2.7}}Q^{\text {tot}}_{\text {rest,LV}} \end{aligned}$$

where $r^{\text {T,i}}$ is the terminal segment radius. An iterative process is used to maintain the values of these baseline flow boundary conditions as much as possible. If they create excessive pressure losses in the tree (and thus too low terminal resistance compared to an ideal minimum terminal resistance), then synthetic trees are dilated to replicate the physiological dilation of arterioles and small arteries. The iterative process for resting conditions is described in detail in ESM section 2.1.

*Hyperemic Conditions* The hyperemic conditions approximate a stress state where the coronary vessels are maximally dilated as occurring when administrating adenosine for invasive FFR measurement or perfusion imaging (ESM section 1). Wilson et al. showed that under these conditions, the total coronary resistance falls to a fourth of the resting value.^43^ Thus, the total ideal hyperemic flow is defined as:

4 $$\begin{aligned} Q_{\text {stress},\text {LV}}^{\text {tot}} = 4 Q_{\text {rest},\text {LV}}^{\text {tot}} \end{aligned}$$

and the diameters of all synthetic segments are dilated to their maximal capacity (40%) from the initial, undilated values, consistent with a Poiseuille relationship between resistance and diameter. Thus, some of the dilation capacity can be used at rest to accommodate oxygen demand of the perfused myocardium at baseline and any remaining dilation capacity is used to simulate maximum hyperemia. Ideal terminal segment flows are calculated as:

5 $$\begin{aligned} q^{\text {T,i}}_{\text {stress}} = 4 q^{\text {T,i}}_{\text {rest}} \end{aligned}$$

The iterative process for hyperemic conditions is described in detail in ESM section 2.2.

*Arteries Not Perfusing the Left Ventricle* For the vessels not perfusing the left ventricle (mostly vessels in proximal RCA), terminal segment flow boundary conditions are:

6 $$\begin{aligned} q^{\text {T,i}}_{\text {rest}} = \frac{(r^{\text {T,i}})^{2.7}}{\sum \nolimits _{i=1}^{n_{\text {term}, \text {non-LV}}} (r^{\text {T,i}})^{2.7}}Q^{\text {tot}}_{\text {rest}, \text {non-LV}} \end{aligned}$$

where $r^{\text {T,i}}$ denotes the terminal segment radius, $n_{\text {term}, \text {non-LV}}$ the number of outlets not perfusing the left ventricle, and $Q^{\text {tot}}_{\text {rest}, \text {non-LV}} =0.2 \cdot Q^{tot}_{\text {rest}, \text {LV}}$ (see Ref. 11). Ideal hyperemic flow is defined as:

7 $$\begin{aligned} q^{\text {tot}}_{\text {stress}, \text {non-LV}} = 4 q^{\text {tot}}_{\text {rest}, \text {non-LV}} \end{aligned}$$

### Myocardium Model

For simplicity, hereafter the left ventricle myocardium and the septum are referred to as the myocardium.

#### Model Description

Blood flow in the myocardium is modeled by a single compartment Darcy model^7^, ^10^:

8a $$\begin{aligned} \varvec{w} + \varvec{K} \nabla p= & {} 0 \end{aligned}$$

8b $$\begin{aligned} \nabla \cdot \varvec{w}= & {} \beta _{\text {source}} \left( p_{\text {source}} - p\right) - \beta _{\text {sink}} \left( p - p_{\text {sink}}\right) \end{aligned}$$

with $\varvec{K}$ the permeability tensor, $\varvec{\omega }$ the Darcy velocity, *p* the capillary bed pressure, $p_{\text {source}}$ and $p_{\text {sink}}$ the source and sink pressure terms respectively, $\beta _{\text {source}}$ and $\beta _{\text {sink}}$ parameters describing the conductance of flow entering and exiting the myocardium respectively.

In terms of physiological meaning, source and sink terms represent respectively the flow entering the myocardium through the coronary model outlets and the venous system drainage. Flow within the myocardium is driven by pressure differences. A no-flux boundary condition is applied to the myocardial wall, which is considered impermeable. Equations 8a and 8b are combined as a single Poisson equation which is solved for *p* using P1 elements, implemented in the FreeFEM framework.^20^ Myocardial meshes consist of $\approx$ 500,000 tetrahedral elements with an average element volume of $2 \times 10^{-4}\,\text {mL}$, a sufficient resolution to obtain mesh-independent solutions with regards to myocardial blood flow.

#### Parameterization

We consider an isotropic permeability field with constant value $K = {2\times 10^{-5}}\text {cm}^{2}\,\text {Pa}^{-1}\text {s}^{-1}$ (see Ref. 7). The coefficient $\beta _{\text {source}}$ is assumed constant over the entire myocardial volume and estimated as:

9 $$\begin{aligned} \beta _{\text {source}} = \frac{Q^{\text {tot}}}{({\overline{p}}_{\text {source}} - {\bar{p}}) V_{\text {LV}}} \end{aligned}$$

where $V_{\text {LV}}$ denotes the myocardial volume, $Q^{\text {tot}}$ the total myocardial blood flow, ${\overline{p}}_{\text {source}}$ the average pressure of all source terms, and ${\bar{p}} = {15}\,\text{mmHg}$ the targeted average capillary pressure.^7^ Notice that the proportionality to $Q^{\text {tot}}$ ensures that $\beta _{\text {source}}$ is increased from rest to hyperemia. This increase reflects the vasodilatory response of the myocardium under hyperemia.

The sink terms are homogeneously distributed over the myocardial volume, thus the coefficient $\beta _{\text {sink}}$ is assumed constant and estimated as:

10 $$\begin{aligned} \beta _{\text {sink}} = \frac{Q^{\text {tot}}}{({\bar{p}} - p_{\text {sink}}) V_{\text {LV}}} \end{aligned}$$

Hence $\beta _{\text {sink}}$ also increases under hyperemia reflecting increased micro-vessel recruitment and venous elastic dilation. $p_{\text {sink}}$ is regarded as the reference pressure and is thus equal to ${0}\,\text {mmHg}$.

### Coupling of the Two Models

The coronary and the myocardium model are strongly coupled. The interaction between the two models takes place at the terminal segment outlets which correspond to the source terms in the porous model description.

Each coronary outlet flow is associated to a respective perfusion territory in the myocardium. Those territories $\Omega _i$ are estimated from a discrete weighted Voronoi tessellation^39^ using the terminal segment diameters as weights (equivalent to a Laguerre tessellation). Consequently, segments with larger diameters, carrying a higher amount of flow, are assigned larger perfusion territories. For blood flow, the coupling process involves an initialization loop, followed by iterations coupling the two models and is described in detail in ESM section 3. Exchange quantities at coupling iteration *k* are illustrated in Fig. 1d. The coupling framework has been developed in an in-house code.

### Post-processing of Results

Simulations provide pressure and flow rate information at different levels. $\text {FFR}_{\text {CT}}$ is computed as the pressure at a given vessel location in the coronary tree divided by aortic inlet pressure. The clinical threshold between positive and negative results is 0.8. Note that for the segmented vasculature, $\text {FFR}_{\text {CT}}$ results are visualized in 3D by projecting centerline values to the arterial wall.

Another quantity of interest is the Myocardial Blood Flow (MBF) for a given perfusion volume, defined as:

11 $$\begin{aligned} \text {MBF}_j = \frac{Q_{j}}{\Omega _{j}} \end{aligned}$$

where $Q_{j}$ is the flow rate associated to a perfusion volume $\Omega _{j}$. When $\Omega _{j}$ corresponds to a Voronoi territory, $Q_{j}$ is the associated terminal segment flow $q^{T,j}$. One can decompose the myocardial volume into smaller volumes and calculate the MBF value of each such volume. Depending on the volume decomposition chosen, characterization of MBF is possible at different spatial resolutions by computing statistics (mean, SD and range) over the MBF values of the sub-volumes.

Here we consider 4 distinct levels of spatial resolution encountered in clinical practice, detailed in Table 2. The 3D simulation results depict MBF values on a scale with characteristic volume of $10^{-4}\,{\text {mL}}$ (”voxels” resolution level in cCTA imaging). The whole myocardial volume is decomposed into 17 standardized segments proposed by the American Heart Association (AHA)^6^ (”AHA segments” resolution level). Based on the AHA segmentation, 3D MBF results are visualized with a 2D perfusion map representing the myocardial volume. Perfusion maps depict MBF values derived from the resolution of the [$^{15}$O]$\text {H}_{{2}}$O PET image, which has a characteristic volume of ${6.4 \times 10^{-2}}\,{\text {mL}}$, while indicating the approximate boundaries of each AHA segment. The apex segment is not included, since its volume is negligible.

The local flow heterogeneity is assessed with the fractal analysis described in Bassingthwaighte *et al*.^3^ The Relative Dispersion ($\text {RD}=\text {SD}/\text {mean}$) values of the flow distribution at each scale are plotted in logarithmic scale against myocardial territory volumes of increasing size. Obtaining a line expresses self-similarity, its slope relating to the Fractal Dimension (FD) as: $\text {slope} = 1 - \text {FD}$. An FD value equal to 1 indicates that the heterogeneity is uniform at all scales. The higher the FD, the more heterogeneous the flow becomes when probed at finer scales.

**Table 2:** Different levels of spatial resolution considered for MBF calculation.

Resolution level	Notation	Total number of volumes	Order of magnitude	Description
Whole myocardium	$\text {MBF}_{\text{myo}}$	1	${100}\,{\text {mL}}$	Organ scale. $\text {MBF}_{\text{myo}}$ is calculated according to Eq. 11, where the entire myocardial volume is considered as the perfusion volume.
Main coronary arteries	$\text {MBF}_{\text{trees}}$	3	${35}\,{\text {mL}}$	Each perfusion volume corresponds to the perfusion territory of each main coronary artery (RCA, LAD, LCX).
AHA segments	$\text {MBF}_{\text{AHA}}$	17	${6}\,{\text {mL}}$	Perfusion volumes correspond to AHA segments detailed in Ref. 6
Voxels	$\text {MBF}_{\text{voxels}}$	$\approx$1,000,000	$10^{-4}\,{\text {mL}}$	High resolution from cCTA, for characterization of MBF at the local level.

## Results

The generated synthetic vasculatures consist of varying number of terminal segments $n_{\text {term}}$ depending on the study. The synthetic trees that did not extend properly during vascular growth due to constraints were trimmed but they carried less than 2% of the total number of terminal segments.

### Baseline Behaviour: Coronary Model vs. Coronary-Myocardium Coupled Model in a Reference Case

We explore the main behavior of the coronary and coupled models by comparing simulation results in both resting and hyperemic conditions for a single patient with non-obstructive CAD (Patient 1). A synthetic vasculature with around 3000 terminal segments defines the reference case. We investigate the main differences between the two models regarding blood flow behaviour in the coronaries and inside the myocardium.

While the mean coronary outlet flow remains the same between the two models, the standard deviation of outlet flows is increased by around 25% for rest and hyperemia in the coupled model compared to the coronary model (Table 3).

**Table 3:** Blood flow results along vasculature and inside the myocardium for vascular networks with varying number of terminal segments for Patient 1.

Number of terminal segments	Coronary model			Coupled model		
	3000	6000	12,000	3000	6000	12,000
Terminal segment flows ([$\text {mL}\,\text {min}^{-1}$]$\times 10^{-2}$)						
Resting conditions	$2.89 \pm 2.25$	$1.44 \pm 1.1$	$0.73 \pm 0.56$	$2.84 \pm 3.62$	$1.42 \pm 2.14$	$0.71 \pm 1.05$
Hyperemic conditions	$9.73 \pm 7.72$	$4.86 \pm 3.79$	$2.44 \pm 1.92$	$9.49 \pm 12.33$	$4.73 \pm 7.31$	$2.36 \pm 3.58$
$\text {MBF}_{\text{AHA}}$ $({\text {mL}\,\text {min}^{-1}\,\text {g}^{-1}})$						
Resting conditions	$1.24 \pm 0.68$ (0.39−3.24)	$1.22 \pm 0.52$ (0.48−2.42)	$1.23 \pm 0.54$ (0.43−2.39)	$1.14 \pm 0.05$ (1.04−1.21)	$1.14 \pm 0.05$ (1.04−1.2)	$1.14 \pm 0.05$ (1.03−1.21)
Hyperemic conditions	$4.17 \pm 2.27$ (1.28−10.32)	$4.11 \pm 1.75$ (1.58−7.75)	$4.12 \pm 1.82$ (1.41−7.77)	$3.84 \pm 0.31$ (3.27−4.32)	$3.83 \pm 0.29$ (3.33−4.28)	$3.79 \pm 0.33$ (3.18−4.3)

**Figure 2:**
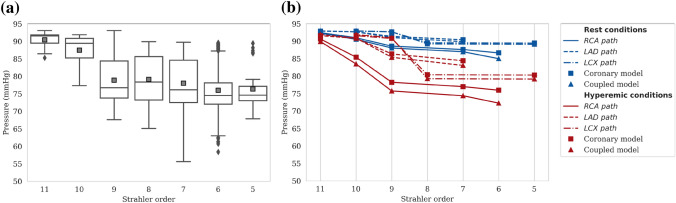
Pressure results along vasculature for reference case. (a) Pressure for vessels in each Strahler order for hyperemic conditions (coupled model). Median and mean pressure values for each order are depicted with horizontal lines and grey squares, respectively. (b) Pressure along 1 random path in each of the three main coronary trees (RCA, LAD and LCX) for resting and hyperemic conditions.

Pressure results along the vasculature are evaluated based on the diameter-defined Strahler order vessel numbering system.^26^ A global view of the pressure drop in hyperemic conditions for the whole vasculature is provided in Fig. 2a, where pressure values for vessels in the same Strahler order are grouped together. While such a description can be useful in identifying median pressure and range at different scales, it can often be misleading: pressure may increase with decreasing order (see median pressure in orders 9–8). For this reason one should not interpret the median, minimum or maximum pressure per order as representative pressure drop along individual paths.

To emphasize this point, pressure drop results for paths spanning from segmented vessels (orders 9–11) to terminal segments (mainly orders 5–7) are plotted individually (Fig. 2b). For visualization purposes, only one randomly selected path per main coronary tree is displayed for both models in resting and hyperemic conditions. As expected, results show a larger pressure drop in hyperemia compared to rest, with minor differences between the two models. Pressure is always decreasing when going down a Strahler order. Similar results were found for all paths.

**Figure 3:**
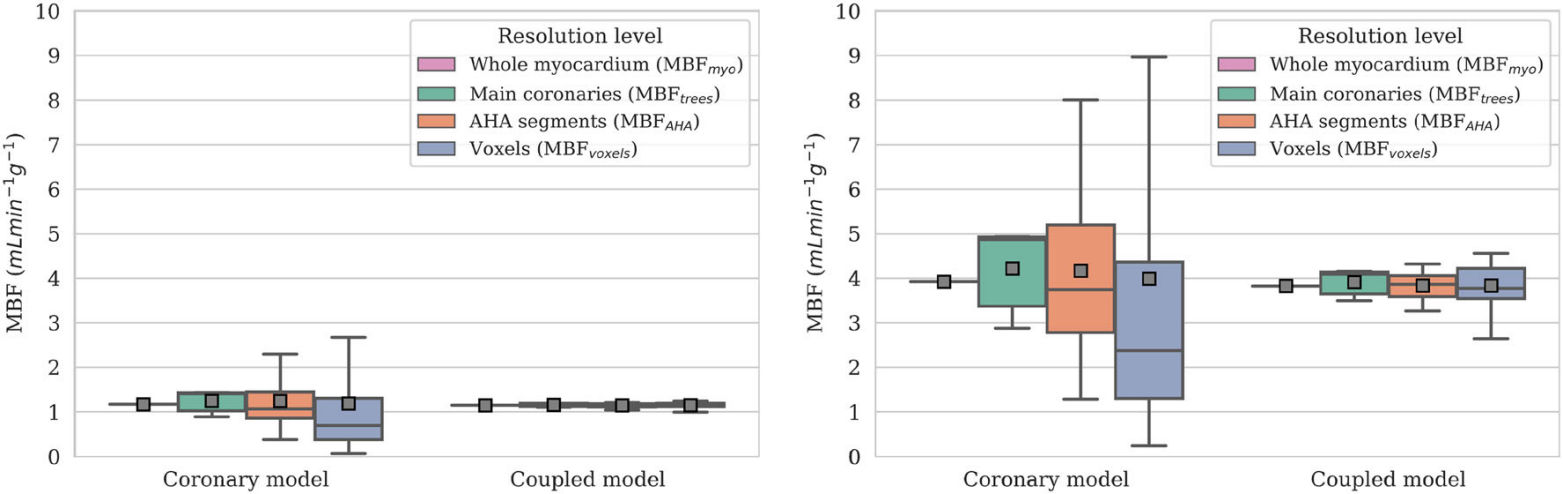
MBF results for the reference case at different levels of spatial resolution detailed in Table 2, for resting (left) and hyperemic (right) conditions. Mean MBF values for each scale are depicted with grey squares. Coronary model outliers for the ”AHA segments” and ”voxels” resolutions are not depicted for legibility purposes; other scales and the coupled model do not present outliers.

Next, we assess blood flow results inside the myocardium. For a given model, mean MBF remains practically constant across the different levels of resolution detailed in Table 2. However, increasing the resolution leads to a significant increase in the standard deviation of MBF values, as locally heterogeneous regions which were previously averaged out are revealed (Fig. 3).

Comparing the two models, no significant difference is observed in predicted mean MBF. However, when taking into account the variance and range of MBF values, the coronary model displays extremely heterogeneous results. On the other hand, the coupled model exhibits a more homogeneous distribution of MBF values which seems physiologically more realistic. In hyperemic conditions, computed mean MBF increases compared to rest by a factor of approximately 3.4 across all scales, reflecting the increase of total flow in the system. The behaviour of the two models remains the same compared to resting conditions.

Finally, the impact of the synthetic vasculature geometry on the results is assessed by generating different vasculatures for Patient 1. In particular, we investigate (1) the effect of the number of terminal segments (ESM section 4.1), and (2) the effect of the inherent randomness in the tree generation method (ESM section 4.2). The coupled model demonstrated robustness in the geometrical variability of the synthetic vasculature for both studies. For the rest of this paper, we generate vasculatures with 12,000 terminal segments in order to explicitly model as many vessels as possible, while maintaining a manageable computational cost.

Thereafter we consider the AHA segment resolution as the default scale for MBF calculation, unless otherwise stated. For simplicity, the AHA subscript is dropped and $\text {MBF}_{\text{AHA}}$ will be denoted by MBF.

### Patients with Non-obstructive CAD

We extend the study to 5 patients (Patient 1 included) with non-obstructive CAD (see ESM section 5 for patient clinical data). Generated vasculatures achieved similar levels of vascular growth for 4 out of 5 patients, extending down to Strahler order 4 (though segments of that order represent $<0.5$% of synthetic segments). Most synthetic segments belong to orders 5 and 6, and constitute 20–30% and 45–55% of the total number of segments, respectively. The vascular network for Patient 5 was the only one that reached a Strahler order of 3 ($<1$% of segments), with 7%, 33% and 41% of synthetic segments belonging to orders 4, 5 and 6, respectively. Diameter distribution within each Strahler order is provided for each patient in ESM section 6.

**Figure 4:**
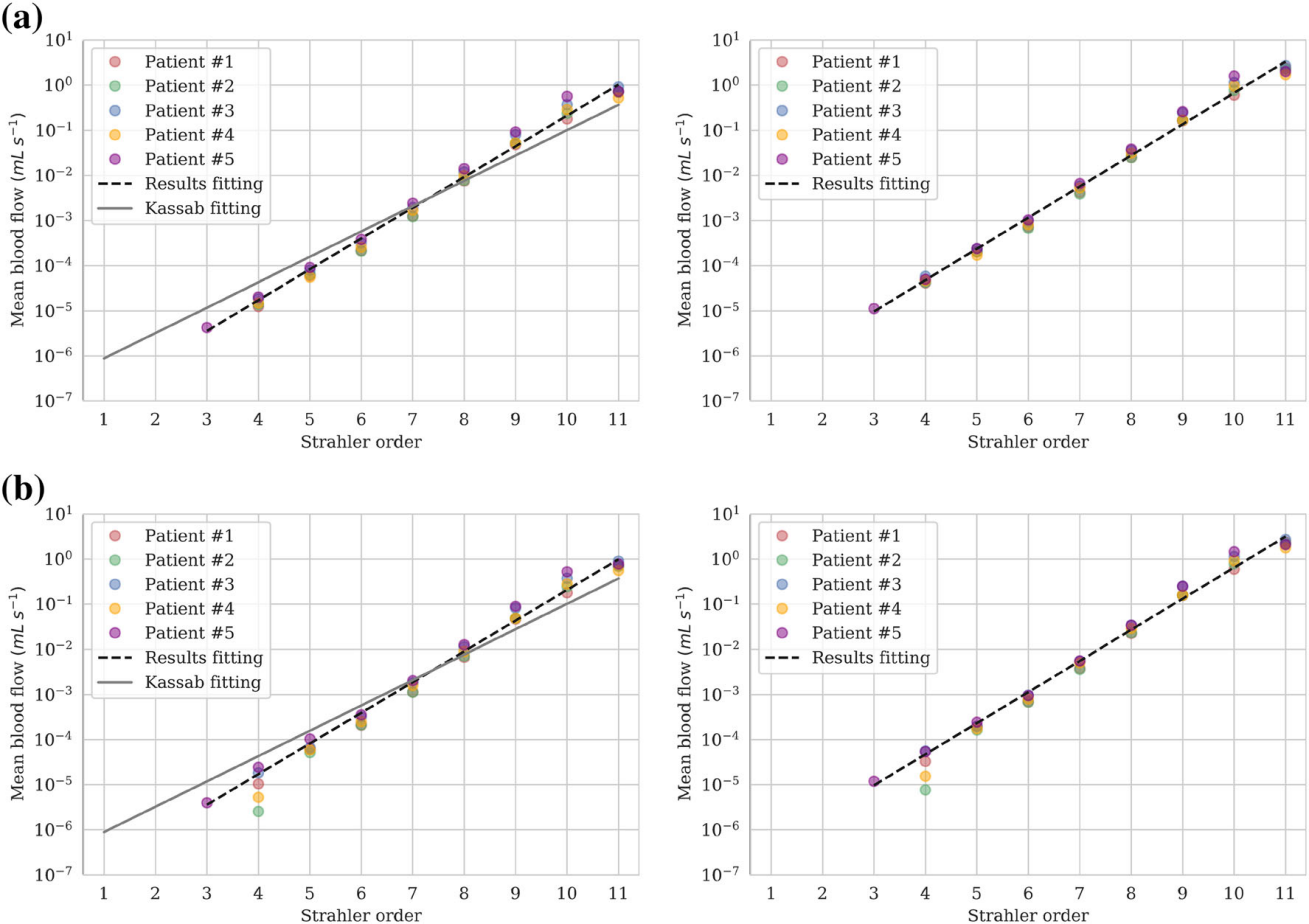
Mean blood flow of elements in the vascular network per Strahler order for (a) coronary model and (b) coupled model. For each model: resting conditions (left), hyperemic conditions (right). The slope of the fitted line for results is equal to 1.57 and 1.59 in resting and hyperemic conditions, respectively, for both models.

*Blood Flow in the Vascular Network* Blood flow results along the vasculature are initially compared to Kassab *et al*.,^25^ where vascular trees were generated based on porcine statistical data. For this purpose, elements (series of segments belonging to the same Strahler order) are grouped by their Strahler order and mean flow for each order is calculated. Coronary model results for resting conditions follow a similar logarithmic law as in Ref. 25 (Fig. 4). The law is maintained in hyperemic conditions with similar slope but higher intercept, as the total flow is increased. Similar results are obtained for the coupled model with the exception of flows at order 4, which deviate from the logarithmic law. This is due to the low amount of segments belonging to that order (with the exception of Patient 5), which does not allow for calculation of reliable statistics.

Findings for the reference case regarding terminal segment flows are extended for the rest of the patients: standard deviation of outlet flows is increased in the coupled model compared to the coronary model, while mean flow remains the same regardless of the model. From resting to hyperemic conditions, the mean outlet flow increase ranges from a factor of 2.7 to 3.35 across patients. The initial target factor of 4 is not attained due to the network resistance. Both conditions have the same relative flow heterogeneity.

Mean pressure drop for the coupled model from the aortic root to tree outlets ranges from 6.5 to 10.6 mmHg at rest, and from 16.5 to 24.2 mmHg at hyperemia for patients 1 to 4, with similar values for the coronary model. Mean pressure drop is higher for Patient 5, as the vasculature extends down to an additional Strahler order, with ${18.7}\,\text {mmHg}$ at rest and ${36.4}\,\text {mmHg}$ at hyperemia. These results are consistent with values obtained in Ref. 25: pressure drop from root to vessels with Strahler order 6, 5 and 4 was reported at 5$\pm {1.3}\,\text {mmHg}$, 8.8$\pm {1.7}\,\text {mmHg}$ and 24.5$\pm {2.8}\,\text {mmHg}$, respectively. To compare, note that for patients 1 to 4 the majority of terminal segments belong to orders 6 and 5, while for Patient 5 to orders 5 and 4. The spatial distribution of pressure highlights the patient-specificity of $\text {FFR}_{\text {CT}}$ analysis (ESM Fig. 6.1).

From resting to hyperemic conditions, mean pressure drop increase ranges from a factor of 1.95 to 2.7 across patients. Note the pressure drop increase is thus less than the increase of flow from rest to hyperemia: this highlights the decrease of tree resistance due to synthetic network dilation, which partially compensates for the resistance increase in the segmented network due to increased flow.

Under resting conditions, the resistance of the overall coronary tree (segmented plus synthetic), defined as $[P_{\text {AO}}- \underset{i}{\text {mean}}(p^{\text {T,i}})]/\underset{i}{\sum }{q^{\text {T,i}}}$, accounts for 7% to 11.3% of the total resistance of the system (macro- and micro-vasculature) for patients 1 to 4 and for 20% for Patient 5. Coronary tree resistance plays a more significant role in hyperemic conditions, making up 17.7% to 26% of total resistance for patients 1 to 4 and 39.1% for Patient 5. The coronary tree resistance is thus non-negligible compared to the resistance of the downstream micro-vasculature. The dilation of the vessels in the synthetic network reduces the network resistance but not enough to compensate for the added inertial resistance of the increased flow from rest to hyperemia.

**Figure 5:**
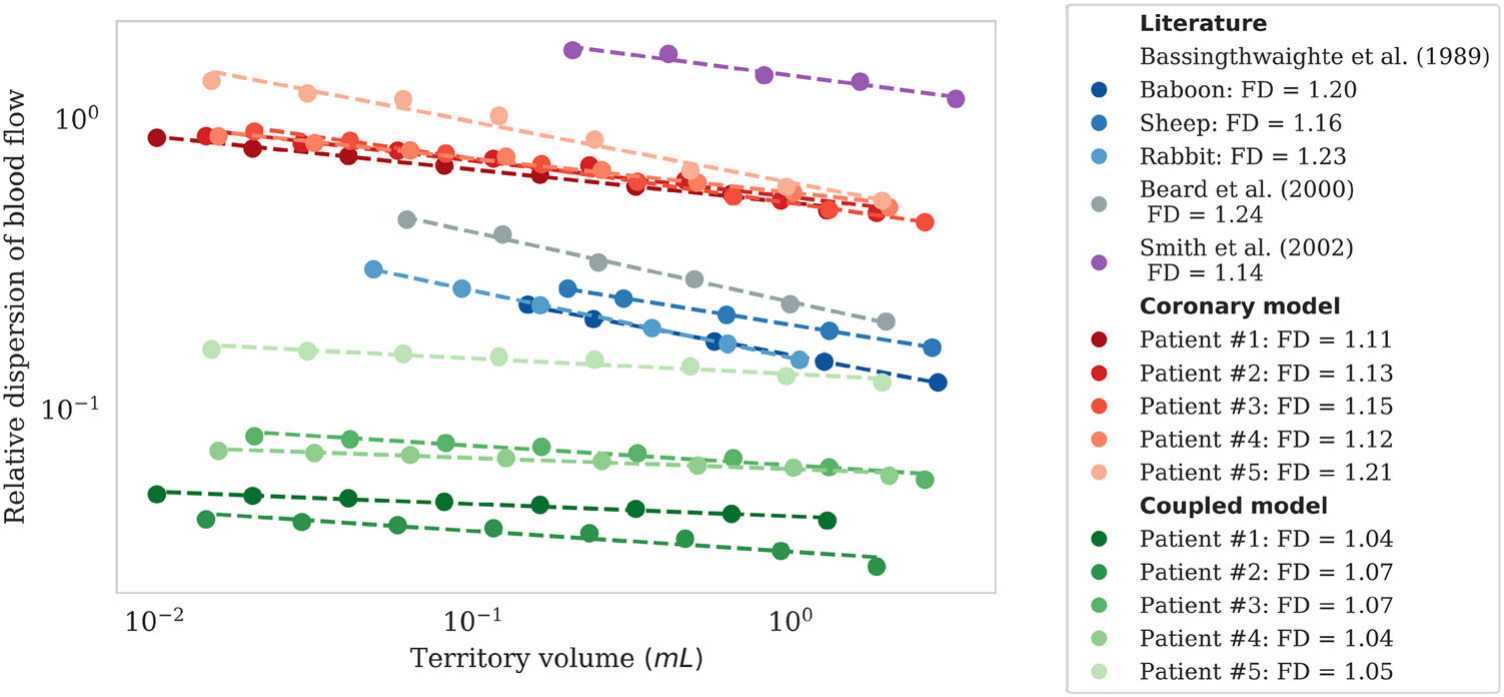
Fractal analysis at resting conditions for 5 patients with non-obstructive CAD and comparison with literature.

*Blood Flow Inside the Myocardium* Following a fractal analysis (described in “Post-processing of results” section), the local flow heterogeneity is assessed under resting conditions for the 5 patients and compared to literature (Fig. 5). Coronary model results are comparable to similar models relying on synthetic networks. In particular, fractal dimension values show good agreement with results obtained with the extended canine synthetic vasculature of Smith *et al*.^41^ The relative dispersion of flow is slightly lower in the coronary model at all scales, indicating a more homogeneous flow distribution. Compared to the synthetic network of Beard *et al*.^4^ based on porcine data, the coronary model exhibits a higher level of flow heterogeneity and lower fractal dimension, but within the same order of magnitude.

The fractal analysis on animal populations by Bassingthwaighte *et al*.^3^ provides reference physiological values. Compared to these data, the coronary model results have consistently higher RD at all scales, while on the contrary, the coupled model consistently lower. This suggests that blood flow predicted by the coronary model tends to be overly heterogeneous, flow predicted by the coupled model overly homogeneous, with physiological values in-between. Furthermore, the very low fractal dimension of the coupled model (close to 1) indicates that there exists only a small difference in heterogeneity across different levels of spatial resolution. The fractal dimension of the coronary model is closer to physiological data.

**Figure 6:**
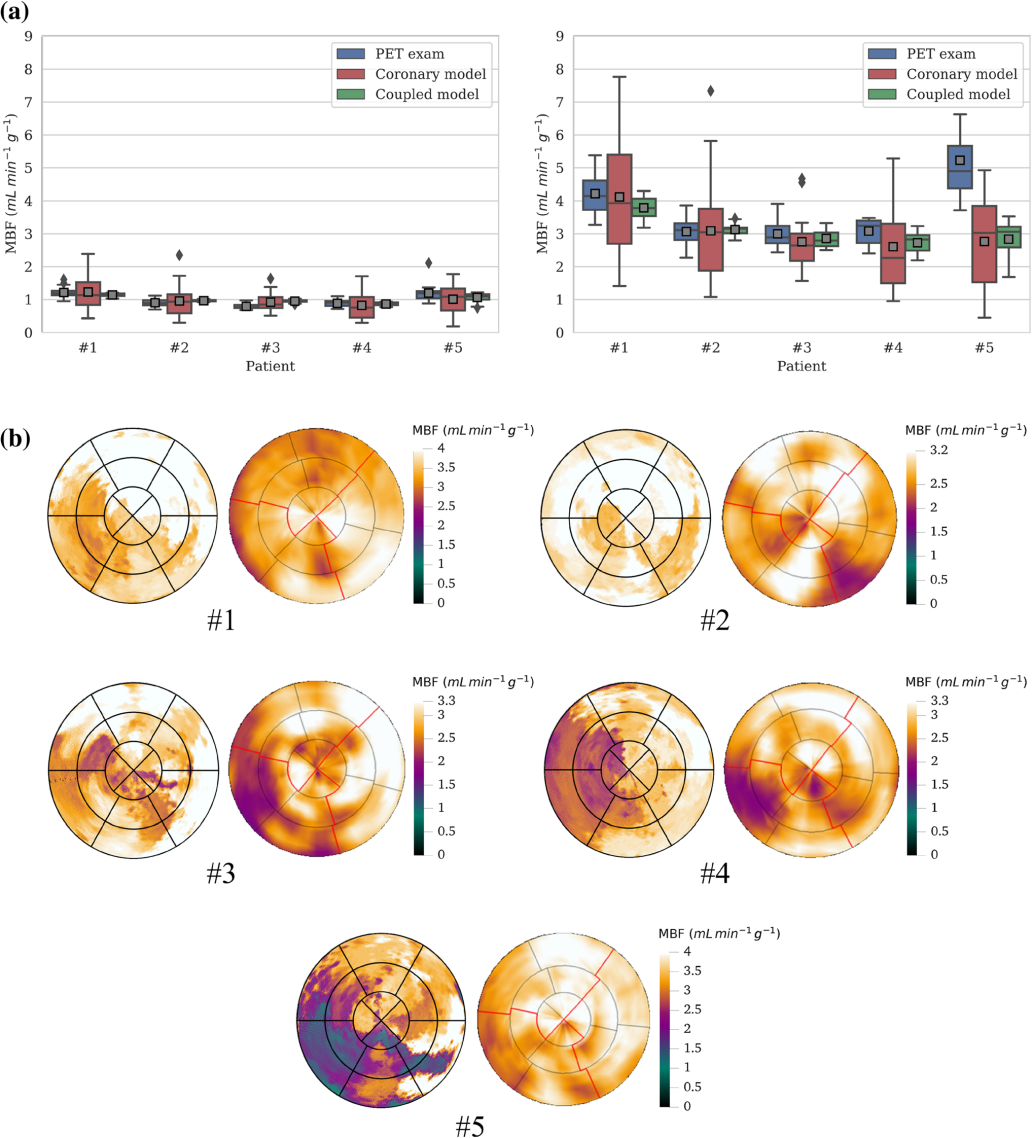
MBF analysis for 5 patients with non-obstructive CAD. (a) Comparison of $\text {MBF}_{\text{AHA}}$ results with [$^{15}$O]$\text {H}_{{2}}$O PET exam data for resting (left) and hyperemic (right) conditions. Mean $\text {MBF}_{\text{AHA}}$ values are depicted with grey squares. (b) Comparison of simulated perfusion maps (left) to [$^{15}$O]$\text {H}_{{2}}$O PET exam perfusion maps (right) for the coupled model under hyperemic conditions. Note the upper limit of color map range is truncated to match PET exam range.

Next, MBF results for both models are compared with [$^{15}$O]$\text {H}_{{2}}$O PET exam patient data (Fig. 6a). For resting conditions, mean MBF matched measured data regardless of the model used, with prediction error less than the mean MBF inter-patient variability. When taking into account the variance of the AHA segment MBF, the coronary model exhibits extremely heterogeneous flow distribution which does not correspond with patient data. This behaviour is consistent with the fractal analysis results. However, the coupled model overcomes this limitation demonstrating MBF values within measured range and better agreement overall. Despite the fact that fractal analysis revealed overly homogeneous flow at smaller scales, the considered spatial resolution is low enough that the model produces physiologically valid results with predictive value. Results are similar for both resting and hyperemic conditions for Patients 1–4.

Simulated perfusion maps for hyperemic conditions are directly compared with [$^{15}$O]$\text {H}_{{2}}$O PET perfusion maps. Coronary model perfusion maps do not correlate well with PET data due to their overly heterogeneous distribution and therefore are not displayed. Coupled model maps (Fig. 6b), while not capturing the exact spatial flow distribution, allow in general for a correct estimation of CAD’s impact on myocardial perfusion. Hyperemic results for Patient 5 did not match measured data, predicting a significant perfusion deficit which was not present in PET data. Lower simulated pressure at the terminal segment outlets led to lower hyperemic MBF. This patient had unique characteristics compared to other patients including higher hyperemic flow and coronary flow reserve (CFR) in the PET exam (Fig. 6a) and a synthetic vasculature upstream of the impaired region that was extended to lower Strahler orders.

### Patient with Obstructive CAD

Here we present preliminary results for an extension of the model to a patient with a lesion in the left anterior descending coronary artery (close to 80% reduction in diameter)—apparent from the $\text {FFR}_{\text {CT}}$ analysis (Fig. 1c). The [$^{15}$O]$\text {H}_{{2}}$O PET exam perfusion map for hyperemic conditions revealed a significant perfusion deficit in the myocardial region corresponding to the LAD tree. For this patient, estimating total coronary flow using the power-law relation between flow and myocardial mass,^8^ led to significantly increased flow compared to PET data (24% and 56% excess flow in resting and hyperemic conditions respectively). For this reason, blood flow in the system was instead estimated with the $\text {MBF}_{\text{myo}}$ value provided by the PET exam in order to determine if the relative effect of the stenosis could be modeled. Previous limitations of the coronary model in terms of flow heterogeneity still apply, thus the analysis is focused on the coupled model.

**Figure 7:**
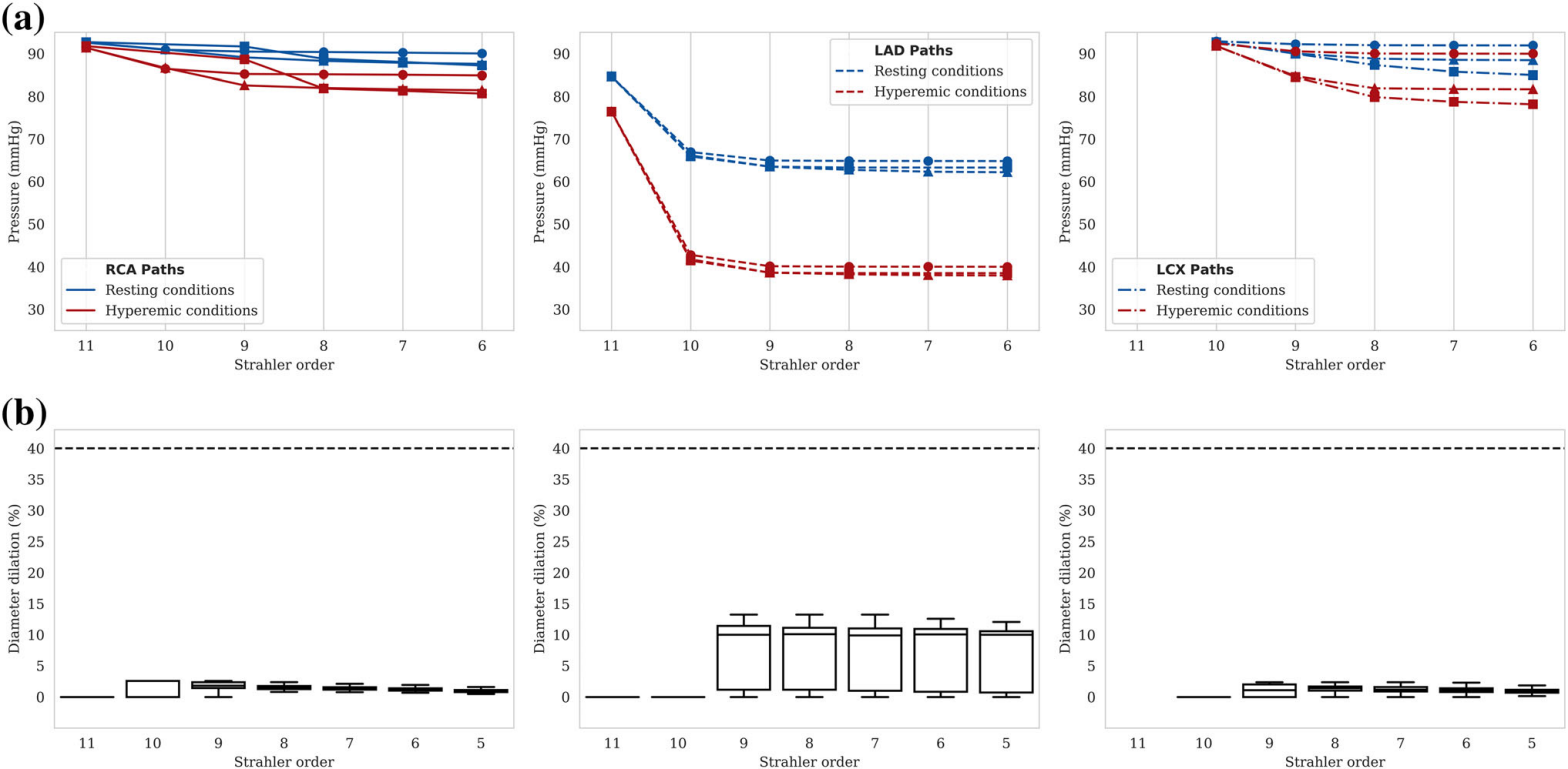
Pressure and dilation analysis along vasculature for a patient with LAD lesion. (a) Pressure along 3 random paths of each main coronary tree for resting and hyperemic conditions. Each path is differentiated with a circle, triangle or square. (b) Diameter dilation per Strahler order in resting conditions for each coronary tree: RCA (left), LAD (center), LCX (right). The horizontal dotted line represents maximum dilation capacity.

As MBF is significantly impaired, this patient provides a suitable framework for evaluation of the model’s capacity for vascular dilation. As demonstrated in Fig. 7b, synthetic segments arising from the LAD were dilated up to 15% under resting conditions, in contrast to healthy trees arising from RCA and LCX where dilation was minimal (up to 3%). These results highlight the model’s ability to account for flow deficits downstream of diseased vessels and adapt diameters accordingly, reflecting the underlying physiological response to ensure adequate myocardial perfusion at rest in patients with epicardial disease.

Pressure drop along three random paths in each main coronary artery is shown in Fig. 7a. LAD paths display higher pressure drop in both resting and hyperemic conditions compared to healthy coronary trees, which takes place almost exclusively in large vessels (between orders 11 and 10). This significant drop early in the vasculature strongly indicates the existence of a hemodynamically significant stenosis in an epicardial vessel of the LAD tree. In addition, outlet pressure decreases almost twice as much from rest to hyperemia in LAD paths ($\approx$ 20 mmHg) compared to RCA and LCX paths ($\approx$ 10 mmHg), reflecting a larger difference between resting and hyperemic flow in regions perfused by LAD trees.

While the total blood flow in the system was explicitly tuned to match PET exam $\text {MBF}_{\text{myo}}$, distribution of flow throughout the coronary tree follows the same procedure as previously described. As illustrated in the perfusion map simulated under hyperemia (Fig. 1f), the exact region of the MBF perfusion deficit in the myocardium (in green) is predicted by the coupled model. Under resting conditions, even though synthetic segments corresponding to the impaired region were dilated (Fig. 7b), a lower perfusion is still visible: the simulated map exhibits the same level of lower perfusion as in the PET exam, but the spatial spread is different. In fact, under hyperemia MBF in the perfusion deficit zone is ${0.92}\,{\text {mL}\,\text {min}^{-1}\text {g}^{-1}}$ for a myocardium average MBF of ${1.9}\,{\text {mL}\,\text {min}^{-1}\text {g}^{-1}}$, which is more drastic than at rest where the lower perfusion is of ${0.73}\,{\text {mL}\,\text {min}^{-1}\text {g}^{-1}}$ for an average MBF of ${0.81}\,{\text {mL}\,\text {min}^{-1}\text {g}^{-1}}$.

## Discussion

In this paper, a multiscale method for simulation of coronary and myocardial blood flow is presented and applied to human data. Patient-specific three-dimensional coronary artery models were segmented using cCTA image data and synthetic trees were generated down to the arteriole level. A stand-alone coronary model and a coronary-myocardium coupled model were investigated with the aim of simulating myocardial perfusion in health and disease. The coupled model demonstrated robustness to variations of the generated vasculature (different initialization, number of terminal segments) and better agreement with [$^{15}$O]$\text {H}_{{2}}$O PET exam data overall. In this section, we discuss some of our modeling assumptions, we propose alternative parameterization choices and provide directions for future work. Limitations and clinical potential of the model are also discussed.

### Model Assumptions and Parameterization

Only steady-state hemodynamic quantities were computed in this paper, which is sufficient for simulated MBF to be comparable to [$^{15}$O]$\text {H}_{{2}}$O PET data, keeping in mind that the acquisition is performed over multiple cardiac cycles. Pulsatile flow simulations would necessitate additional assumptions, more parameters to estimate,^30^ or other patient-specific data not easily measurable, overall introducing additional complexity which is outside of the scope of the current study. In future work, incorporation of flow pulsatility in the model would potentially allow for assessing the risk of plaque rupture in the coronaries.

Moreover, blood was modeled as a Newtonian fluid. For larger epicardial vessels segmented from cCTA, shear rate was above ${10}\,\text {s}^{-1}$ for all patients in this study. Recent 3D work^1^ has demonstrated for mild to severe coronary stenoses that the Newtonian model did not lead to a significantly different centerline velocity compared to three non-Newtonian models, validated by clinical measurements. Moreover, while viscosity changes can affect velocity profiles, it has a minor effect on flow and pressure losses.^38^ The effect of viscosity uncertainty on perfusion could be assessed in future work, but it would require additional patient-specific measurements (hematocrit, plasma viscosity). For smaller synthetic vessels (Strahler orders 4–5), future work could also consider the Fahraeus-Lindqvist effect and plasma skimming.

A single Darcy compartment was used in the myocardium model with homogeneous flow conductance parameters ($\beta _{\text {source}}$ and $\beta _{\text {sink}}$). Previous studies have considered more complex porous model descriptions with multiple compartments modeling different scales of the micro-circulation,^21^, ^31^ but this approach makes the flow conductance parameters difficult to estimate and relies on complex anatomical information. The simpler myocardium model proposed herein enables parameter estimation using easier-to-derive patient-specific variables, such as the myocardial volume and the total coronary flow, for which estimation laws already exist.^8^, ^42^ This simplified approach is sufficient for MBF characterization at the AHA segment level as demonstrated by the good agreement with PET exam data. However, finer details of the flow are not captured which can be seen in the almost uniform flow at lower spatial resolutions (Fig. 5). This could be tackled with a homogenization approach on parameter estimation.^22^

Several refinements in the parameterization of the coupled model are possible. While the mechanical properties of the myocardial tissue are non-uniform and anisotropic,^7^ the myocardial permeability tensor $\varvec{K}$ is considered herein as constant and isotropic. Note that simulations with an increased *K* value, to account for the additional vessels recruited under hyperemia,^19^ did not result in any noticeable difference in terms of perfusion. Furthermore, the aortic pressure is based on a population average. Patient-specific values could help fine-tune the range and spatial distribution of MBF for each patient but are variable at rest and difficult to predict under hyperemic conditions.

As shown in the simulated perfusion maps, even though the main MBF characteristics are adequately captured, the spatial distribution of flow does not exactly match PET exam maps. Note that for any given MBF distribution, a corresponding source-sink field can be determined and vice versa. An interesting direction for future work to improve results such as for Patient 5, would be to estimate spatially-heterogeneous flow conductance parameters via a machine learning model, utilizing PET exam data and other patient-specific features.

### Limitations

In the current model, total flow in hyperemic conditions cannot exceed four times the resting total flow. As a result, the model is unable to predict MBF of patients with CFR higher than 4, like Patient 5. Interestingly for this patient, in a separate simulation with increased hyperemic total flow based on PET data, a perfusion deficit with similar magnitude is still present. This indicates that the upstream vasculature is unable to accommodate additional flow, which could be a potential drawback of the synthetic network generation method.

Challenges with the estimation of total flow are apparent from the example of the patient with a severe LAD lesion as the myocardial-mass-based total flow over-estimated the baseline flow. While there is significant variability in resting MBF,^13^ our current estimation method is able to capture the reported median level of resting MBF for patients with non-obstructive CAD, as seen in Fig. 6a (rest). For the obstructive CAD patient, our method overestimated total flow by 24%, which is nevertheless within the range reported by Danad *et al*. for obstructive CAD patients. Studies in additional patients with obstructive CAD may be needed to determine if parameters in addition to the myocardial mass are needed to predict absolute resting blood flow in such patients. Note, in separate simulations, the coupled model was still able to identify the perfusion deficit region, even when the flow estimation was based on myocardial mass, albeit with over-estimated absolute MBF values.

Results on a patient with LAD stenosis demonstrated the model’s ability to mimic the physiological response of arterial dilation. While vasculature downstream of a diseased epicardial vessel was partially dilated at rest to accommodate more flow, the spatial results show that, as for some of non-obstructive CAD patients, the model may need to include more heterogeneous parameterization.

Future work will include a study on how the coupled model affects the prediction of $\text {FFR}_{\text {CT}}$, especially for cases near the cut-off value. Finally, more patients are needed for further validation of the model.

### Conclusion and Perspectives on Clinical Relevance

A full pipeline from cCTA to simulation of MBF with potential for clinical application is described. Absolute hyperemic MBF has been shown to have better prognostic^5^ and diagnostic^13^ performances compared to other clinical metrics, like CFR. By utilizing readily available information from a CT scan, this approach enables quantitative assessment of CAD’s impact on myocardial perfusion. With the exception of one non-obstructive CAD case for which the model needs to be further refined, simulated MBF matched [$^{15}$O]$\text {H}_{{2}}$O PET exam data at the AHA segment scale, a widely used resolution in clinical practice. In case of severe CAD, a direct link between coronary artery narrowing and impaired myocardial blood flow is achieved. This framework also makes possible the calculation of ischemic burden, an important clinical metric for evaluation of CAD severity. At the same time, it provides a testbed to explore different combinations of positive/negative FFR and presence/absence of perfusion deficit and better elucidate their different contributions for treatment choice.

As $\text {FFR}_{\text {CT}}$ analysis is already utilized in clinical practice, incorporation of this method could enhance its current capabilities by extending the analysis to the myocardium: it would constitute a diagnostic tool capable of multiscale simulation of blood flow from the epicardial coronary arteries to the myocardial tissue and enable identification of those regions of the myocardium with diminished blood flow or at risk of infarction in the event of atherosclerotic plaque rupture.

## Electronic supplementary material

Below is the link to the electronic supplementary material.

Electronic supplementary material 1 (PDF 22758 kb).

## References

[1] Abbasian M, Shams M, Valizadeh Z, Moshfegh A, Javadzadegan A, Cheng S (2020). Effects of different non-newtonian models on unsteady blood flow hemodynamics in patient-specific arterial models with in-vivo validation. Comput. Methods Programs Biomed..

[2] Alves JR, de Queiroz RA, Bar M, dos Santos RW (2019). Simulation of the perfusion of contrast agent used in cardiac magnetic resonance: A step toward non-invasive cardiac perfusion quantification. Front. Physiol..

[3] Bassingthwaighte JB, King RB, Roger SA (1989). Fractal nature of regional myocardial blood flow heterogeneity. Circ. Res..

[4] Beard DA, Bassingthwaighte JB (2000). The fractal nature of myocardial blood flow emerges from a whole-organ model of arterial network. J. Vasc. Res..

[5] Bom MJ, van Diemen PA, Driessen RS, Everaars H, Schumacher SP, Wijmenga J-T, Raijmakers PG, van de Ven PM, Lammertsma AA, van Rossum AC, Knuuti J, Danad I, Knaapen P (2019). Prognostic value of [15O]H2O positron emission tomography-derived global and regional myocardial perfusion. Eur. Heart J. Cardiovasc. Imaging.

[6] Cerqueira MD, Weissman NJ, Dilsizian V, Jacobs AK, Kaul S, Laskey WK, Pennell DJ, Rumberger JA, Ryan T, Verani MS (2002). Standardized myocardial segmentation and nomenclature for tomographic imaging of the heart A statement for healthcare professionals from the Cardiac Imaging Committee of the Council on Clinical Cardiology of the American Heart Association. Int. J. Cardiovasc. Imaging.

[7] Chapelle D, Gerbeau J-F, Sainte-Marie J, Vignon-Clementel I (2010). A poroelastic model valid in large strains with applications to perfusion in cardiac modeling. Comput. Mech..

[8] Choy JS, Kassab GS (2008). Scaling of myocardial mass to flow and morphometry of coronary arteries. J. Appl. Physiol..

[9] Chung J-H, Lee KE, Nam C-W, Doh J-H, Kim HI, Kwon S-S, Shim EB, Shin E-S (2017). Diagnostic performance of a novel method for fractional flow reserve computed from noninvasive computed tomography angiography (NOVEL-FLOW study). The American journal of cardiology.

[10] Cookson A, Lee J, Michler C, Chabiniok R, Hyde E, Nordsletten D, Sinclair M, Siebes M, Smith N (2012). A novel porous mechanical framework for modelling the interaction between coronary perfusion and myocardial mechanics. J. Biomech..

[11] Crystal GJ, Pagel PS (2018). Right ventricular perfusion: physiology and clinical implications. Anesthesiology.

[12] Danad I, Raijmakers PG, Driessen RS, Leipsic J, Raju R, Naoum C, Knuuti J, Mki M, Underwood RS, Min JK, Elmore K, Stuijfzand WJ, van Royen N, Tulevski II, Somsen AG, Huisman MC, van Lingen AA, Heymans MW, van de Ven PM, van Kuijk C, Lammertsma AA, van Rossum AC, Knaapen P (2017). Comparison of coronary CT angiography, SPECT, PET, and hybrid imaging for diagnosis of ischemic heart disease determined by fractional flow reserve. JAMA Cardiology.

[13] Danad I, Uusitalo V, Kero T, Saraste A, Raijmakers PG, Lammertsma AA, Heymans MW, Kajander SA, Pietil M, James S, Srensen J, Knaapen P, Knuuti J (2014). Quantitative assessment of myocardialperfusion in the detection of significant coronary artery disease: Cutoff values and diagnostic accuracy of quantitative [15O]H2O pet imaging. J. Am. Coll. Cardiol..

[14] de Bruyne B, Bartunek J, Sys SU, Pijls NH, Heyndrickx GR, Wijns W (1996). Simultaneous coronary pressure and flow velocity measurements in humans. Circulation.

[15] Driessen RS, Danad I, Stuijfzand WJ, Raijmakers PG, Schumacher SP, van Diemen PA, Leipsic JA, Knuuti J, Underwood SR, van de Ven PM, van Rossum AC, Taylor CA, Knaapen P (2019). Comparison of coronary computed tomography angiography, fractional flow reserve, and perfusion imaging for ischemia diagnosis. JACC.

[16] Ernest WC, Lo RT, Menezes LJ (2020). On outflow boundary conditions for CT-based computation of FFR: Examination using PET images. Med. Eng. Phys..

[17] Formaggia L, Lamponi D, Quarteroni A (2003). One-dimensional models for blood flow in arteries. J. Eng. Math..

[18] Fossan FE, Sturdy J, Muller LO, Strand A, Braaten AT, Jorgensen A, Wiseth R, Hellevik LR (2018). Uncertainty quantification and sensitivity analysis for computational FFR estimation in stable coronary artery disease. Cardiovascular engineering and technology.

[19] Goodwill, A. G., G. M. Dick, A. M. Kiel, and J. D. Tune. Regulation of Coronary Blood Flow. American Cancer Society, Washington pp. 321--382 (2017). 10.1002/cphy.c160016 PMC5966026 28333376

[20] Hecht F (2012). New development in FreeFem++. J. Numer. Math..

[21] Hyde ER, Cookson AN, Lee J, Michler C, Goyal A, Sochi T, Chabiniok R, Sinclair M, Nordsletten DA, Spaan J, van den Wijngaard JPHM, Siebes M, Smith NP (2014). Multi-scale parameterisation of a myocardial perfusion model using whole-organ arterial networks. Ann. Biomed. Eng..

[22] Hyde, E. R., C. Michler, J. Lee, A. N. Cookson, R. Chabiniok, D. A. Nordsletten, and N. P. Smith. Parameterisation of multi-scale continuum perfusion models from discrete vascular networks. *Med. Biol. Eng. Comput.* 51:557--570, 2013. 10.1007/s11517-012-1025-2 PMC3627025 23345008

[23] Jaquet C, Najman L, Talbot H, Grady L, Schaap M, Spain B, Kim HJ, Vignon-Clementel I, Taylor CA (2019). Generation of patient-specific cardiac vascular networks: a hybrid image-based and synthetic geometric model. IEEE Trans. Biomed. Eng..

[24] Johnson, N. P., D. T. Johnson, R. L. Kirkeeide, C. Berry, B. D. Bruyne, W. F. Fearon, K. G. Oldroyd, N. H. Pijls, and K. L. Gould. Repeatability of fractional flow reserve despite variations in systemic and coronary hemodynamics. *JACC Cardiovasc. Interv.* 8:1018--1027, 2015. 10.1016/j.jcin.2015.01.039 26205441

[25] Kassab GS, Berkley J, Fung Y-CB (1997). Analysis of pigs coronary arterial blood flow with detailed anatomical data. Ann. Biomed. Eng..

[26] Kassab GS, Rider CA, Tang NJ, Fung YC (1993). Morphometry of pig coronary arterial trees. Am. J. Physiol.-Heart Circ. Phys..

[27] Kim H, Vignon-Clementel I, Coogan J, Figueroa C, Jansen K, Taylor C (2010). Patient-specific modeling of blood flow and pressure in human coronary arteries. Ann. Biomed. Eng..

[28] Koo B-K, Erglis A, Doh J-H, Daniels DV, Jegere S, Kim H-S, Dunning A, DeFrance T, Lansky A, Leipsic J, Min JK (2011). Diagnosis of ischemia-causing coronary stenoses by noninvasive fractional flow reserve computed from coronary computed tomographic angiograms: results from the prospective multicenter DISCOVER-FLOW (Diagnosis of Ischemia-Causing Stenoses Obtained Via Noninvasive Fractional Flow Reserve) study. J. Am. Coll. Cardiol..

[29] Lee, J., A. Cookson, R. Chabiniok, S. Rivolo, E. Hyde, M. Sinclair, C. Michler, T. Sochi, and N. Smith. Multiscale modelling of cardiac perfusion. In: A. Quarteroni (ed) Modeling the Heart and the Circulatory System, pp. 51--96, Springer, New York (2015).

[30] Lee J, Nordsletten D, Cookson A, Rivolo S, Smith N (2016). In silico coronary wave intensity analysis: application of an integrated one-dimensional and poromechanical model of cardiac perfusion. Biomech. Model. Mechanobiol..

[31] Michler C, Cookson AN, Chabiniok R, Hyde E, Lee J, Sinclair M, Sochi T, Goyal A, Vigueras G, Nordsletten DA, Smith NP (2013). A computationally efficient framework for the simulation of cardiac perfusion using a multi-compartment darcy porous-media flow model. International journal for numerical methods in biomedical engineering.

[32] Min JK, Leipsic J, Pencina MJ, Berman DS, Koo BK, van Mieghem C, Erglis A, Lin FY, Dunning AM, Apruzzese P, Budoff MJ, Cole JH, Jaffer FA, Leon MB, Malpeso J, Mancini GB, Park SJ, Schwartz RS, Shaw LJ, Mauri L (2012). Diagnostic accuracy of fractional flow reserve from anatomic CT angiography. JAMA.

[33] Muller, L. O., F. E. Fossan, A. T. Braaten, A. Jorgensen, R. Wiseth, and L. R. Hellevik. Impact of baseline coronary flow and its distribution on fractional flow reserve prediction. *Int. J. Num. Methods Biomed. Eng.* 9, 3246, 2019. 10.1002/cnm.3246 31397083

[34] Nakazato, R., H.-B. Park, D. S. Berman, H. Gransar, B.-K. Koo, A. Erglis, F. Y. Lin, A. M. Dunning, M. J. Budoff, J. Malpeso, J. Leipsic, and J. K. Min. Noninvasive fractional flow reserve derived from computed tomography angiography for coronary lesions of intermediate stenosis severity: results from the DeFACTO study. *Circ. Cardiovasc. Imaging* 6:881–889, 2013. 10.1161/CIRCIMAGING.113.000297 24081777

[35] Norgaard BL, Leipsic J, Gaur S, Seneviratne S, Ko BS, Ito H, Jensen JM, Mauri L, De Bruyne B, Bezerra H, Osawa K, Marwan M, Naber C, Erglis A, Park SJ, Christiansen EH, Kaltoft A, Lassen JF, Botker HE, Achenbach S (2014). Diagnostic performance of noninvasive fractional flow reserve derived from coronary computed tomography angiography in suspected coronary artery disease: the NXT trial (analysis of coronary blood flow using CT angiography: Next steps). JACC.

[36] Patel MR, Norgaard BL, Fairbairn TA, Nieman K, Akasaka T, Berman DS, Raff GL, HurwitzKoweek LM, Pontone G, Kawasaki T, Sand NP, Jensen JM, Amano T, Poon M, Vrehus KA, Sonck J, Rabbat MG, Mullen S, De Bruyne B, Rogers C, Matsuo H, Bax JJ, Leipsic J (2020). 1-year impact on medical practice and clinical outcomes of FFRCT The ADVANCE registry. JACC Cardiovasc Imaging.

[37] Patel MR, Peterson ED, Dai D, Brennan JM, Redberg RF, Anderson HV, Brindis RG, Douglas PS (2010). Low diagnostic yield of elective coronary angiography. N. Engl. J. Med..

[38] Sankaran S, Kim HJ, Choi G, Taylor CA (2016). Uncertainty quantification in coronary blood flow simulations: Impact of geometry, boundary conditions and blood viscosity. J. Biomech..

[39] Serra J (1982). Image analysis and mathematical morphology,.

[40] Sharma, P., L. M. Itu, X. Zheng, A. Kamen, D. Bernhardt, C. Suciu, and D. Comaniciu. A framework for personalization of coronary flow computations during rest and hyperemia. *IEEE Eng. Med. Biol. Soc.* 11: 6665--6668, 2012. 10.1109/EMBC.2012.6347523 23367458

[41] Smith N, Pullan A, Hunter PJ (2002). An anatomically based model of transient coronary blood flow in the heart. SIAM Journal on Applied mathematics.

[42] Taylor CA, Fonte TA, Min JK (2013). Computational fluid dynamics applied to cardiac computed tomography for noninvasive quantification of fractional flow reserve: scientific basis. J. Am. Coll. Cardiol..

[43] Wilson RF, Wyche K, Christensen BV, Zimmer S, Laxson DD (1990). Effects of adenosine on human coronary arterial circulation. Circulation.

[44] Zhang Y, Barocas VH, Berceli SA, Clancy CE, Eckmann DM, Garbey M, Kassab GS, Lochner DR, McCulloch AD, Tran-Son-Tay R, Trayanova NA (2016). Multi-scale modeling of the cardiovascular system: disease development, progression, and clinical intervention. Ann. Biomed. Eng..

