# Supplementary material for: Myocardial Perfusion Simulation for Coronary Artery Disease: A Coupled Patient-Specific Multiscale Model
Source: Ann Biomed Eng. 2020 Dec 1;49(5):1432–47. doi: 10.1007/s10439-020-02681-z (PMC8057976; doi:10.1007/s10439-020-02681-z)
Supplement: Supplementary file 1 — Electronic supplementary material 1 (PDF 22758 kb). [file 10439_2020_2681_MOESM1_ESM.pdf]

Electronic Supplementary Material (ESM)

1 Imaging protocols

The imaging data for coronary artery and myocardium segmentation from cCTA and for perfusion map ground truth from $[^{15}O]H_2O$ PET was acquired as described by Danad et. al.[1].

1.1 Coronary Computed Tomography Angiography (cCTA)

Patients underwent coronary angiography on a 256-slice CT scanner (Philips Brilliance iCT, Philips Healthcare, Best, the Netherlands) with a collimation 128 x 0.625 mm and a tube rotation time of 270 ms. To visualize the coronary artery lumen a bolus of 100 mL iobitidol (Xenetix 350) was injected intravenously $5.7\,\mathrm{mL\,s^{-1}}$, with immediately after a 50 mL saline chaser. The scan was triggered with an automatic bolus tracking technique. The region of interest was placed in the descending thoracic aorta with a threshold of 150 HU. Metoprolol 50 to 150 mg was administered orally if patients had a prescan HR $\geq$ 65 beats per minute (bpm) one hour before the start of the CT protocol. If necessary, 5 to 25 mg metoprolol was given intravenously during the scan to achieve a heart rate $<$ 65 bpm. All patients received 800 µg of sublingual nitroglycerine immediately before cCTA.

1.2 $[^{15}O]H_2O$ Positron Emission Tomography (PET)

Patients had to refrain from taking products containing caffeine or xanthine 24 hours before imaging. Patients fasted for at least 4 hours before the scan protocol. All patients were imaged on a hybrid PET/CT device (Philips Gemini TF 64, Philips Healthcare, Best, The Netherlands). During resting conditions as well as vasodilator stress induced by intravenous infusion of adenosine ($140\,\mathrm{\mu g\,kg^{-1}\,min^{-1}}$), a 370 MBq of $[^{15}O]H_2O$ was used as a perfusion tracer. For more information on the cardiac $[^{15}O]H_2O$ PET protocol, image acquisition and quantification of MBF, see Danad et al.[2].

2 Parameterization of coronary model in resting and hyperemic conditions: Detailed description

2.1 Resting conditions

Using the initialized flows in eq. 3 (main paper), we calculate ideal baseline resistance at each terminal segment:

$$R_{\text{base}}^{\text{T,i}} = \frac{P_{\text{AO}}}{q_{\text{rest}}^{\text{T,i}}} \tag{1}$$

For each terminal segment, an expected minimum resistance is estimated as follows:

$$R_{\text{min}}^{\text{T,i}} = \frac{1}{4} R_{\text{base}}^{\text{T,i}} \tag{2}$$

Here, we assume a uniform factor but this can vary for each patient. Anatomically, the factor represents a maximal radius dilation capacity of 40% based on uniform dilation of a tree to achieve a 4-fold reduction in resistance[5]. Note that minimum resistances are calculated once and fixed for the whole parameterization process.

Using the terminal segment flows as boundary conditions for the first iteration, flow and pressure are computed for the entire tree by solving the 1D equations. The computed terminal segment pressure $p^{\text{T,i}}$ and the assigned terminal segment flow $q_{\text{rest}}^{\text{T,i}}$ determine the simulated terminal segment resistance $R_{\text{sim}}^{\text{T,i}}$:

$$R_{\text{sim}}^{\text{T,i}} = \frac{p^{\text{T,i}}}{q_{\text{rest}}^{\text{T,i}}} \tag{3}$$

If the simulated terminal segment resistance is lower than the minimum resistance value $R_{\text{min}}^{\text{T,i}}$, we update both the geometry and the flow. We dilate the radius of the terminal segment and its upstream synthetic segments by multiplying them with a uniform factor of $\left(\frac{R_{\text{base}}^{\text{T,i}}}{R_{\text{min}}^{\text{T,i}}}\right)^{0.25} = \sqrt{2}$. We reduce the terminal segment flow by δq by solving the following equation

$$(q_{\text{sim,rest}}^{\text{T,i}} + \delta q) R_{\text{min}}^{\text{T,i}} = P_{AO} - R_p^{\text{T,i}} (q_{\text{sim,rest}}^{\text{T,i}} + \delta q) \tag{4}$$

where $R_p^{\text{T,i}} = \frac{P_{\text{AO}} - p_{\text{sim,rest}}^{\text{T,i}}}{q_{\text{sim,rest}}^{\text{T,i}}}$.

If the simulated terminal segment resistance is greater or equal to $R^{\mathrm{T,i}}_{\mathrm{min}}$, we update the geometry by multiplying by a factor of $\left(\frac{R^{\mathrm{T,i}}_{\mathrm{base}}}{R^{\mathrm{T,i}}_{sim}}\right)^{0.25}$ but maintain the same flow $q^{\mathrm{T,i}}_{\mathrm{rest}}$.

Note that each terminal segment can have a different radius dilation factor as the resistance of the terminal segment will depend on the pressure loss accumulated along the path. For the upstream segments branching to child segments with different dilation factors, bigger dilation factors are chosen to dilate them. Several iterations are necessary to obtain convergence as both the geometry and boundary conditions are updated in each iteration. The convergence criteria used:

$$\max_i \left| \frac{R^{\mathrm{T,i}}_{\mathrm{sim,n+1}} - R^{\mathrm{T,i}}_{\mathrm{sim,n}}}{R^{\mathrm{T,i}}_{\mathrm{sim,n+1}}} \right| < 1\% \tag{5}$$

with n the iteration counter. Note that for synthetic trees arising from the segmented vessel outlets, the dilation propagation stops before reaching the root segment since this was directly observed in the CT data. For synthetic trees arising along a segmented vessel, the dilation propagation is applied up to and including the root segment. This ensures that only arteriole and small artery vessels are dilated since they contribute most to coronary resistance modulation and are dilated most under hyperemic conditions. Further, the CT data is obtained with the use of sublingual nitrates so the segmented tree is assumed to be fully dilated at rest.

2.2 Hyperemic conditions

The minimum resistance at each terminal segment is computed using the ideal terminal segment flow:

$$R^{T,i}_{\mathrm{min,stress}} = \frac{P_{\mathrm{AO}}}{q^{\mathrm{T,i}}_{\mathrm{stress}}} \tag{6}$$

Since the whole system is already dilated to the maximum radius dilation capacity, only terminal flows can be changed. The system is first solved using $q^{\mathrm{T,i}}_{\mathrm{stress}}$ as a boundary condition at each terminal segment outlet. With the resulting simulated pressures $p^{\mathrm{T,i}}_{\mathrm{stress}}$ and set flows, we calculate the simulated resistance:

$$R^{T,i}_{\mathrm{sim,stress}} = \frac{p^{\mathrm{T,i}}_{\mathrm{stress}}}{q^{\mathrm{T,i}}_{\mathrm{stress}}} \tag{7}$$

In each iteration, the terminal segment flow is incremented by δq by solving the following equation:

$$(q^{\mathrm{T,i}}_{\mathrm{sim,stress}} + \delta q) R^{\mathrm{T,i}}_{\mathrm{min,\ stress}} = P_{\mathrm{AO}} - R_p^{\mathrm{T,i}} (q^{\mathrm{T,i}}_{\mathrm{sim,stress}} + \delta q) \tag{8}$$

where $R_p^{\mathrm{T,i}} = \frac{P_{\mathrm{AO}} - p^{\mathrm{T,i}}_{\mathrm{sim,stress}}}{q^{\mathrm{T,i}}_{\mathrm{sim,stress}}}$.

Consequently when the simulated resistance $R^{\mathrm{T,i}}_{\mathrm{sim,stress}}$ is less than the minimum resistance $R^{\mathrm{T,i}}_{\mathrm{min,stress}}$, the flow is decreased. Otherwise flow is increased.

Simulation is repeated with the updated terminal flow values. Upon iteration, the computed terminal resistance values converge to the minimum resistance values. The convergence criteria is:

$$\max_i \left| \frac{R^{\mathrm{T,i}}_{\mathrm{sim,stress,n}} - R^{\mathrm{T,i}}_{\mathrm{min,stress}}}{R^{\mathrm{T,i}}_{\mathrm{min,stress}}} \right| < 1\% \tag{9}$$

3 Coupling method

For blood flow, the coupling process involves an initialization loop, followed by iterations coupling the two models. The variable k represents the coupling iteration counter.

During the initialization loop ($k = 0$), 1D equations are solved iteratively to generate possibly dilated geometry with converged flow and pressure fields as described in the parameterization section, either for rest or hyperemic conditions. Initial porous model parameters are determined using the converged flow and pressure values. The converged outlet pressures $p^{\mathrm{T,i}}_{k=0}$ are used to estimate the β_{source} and β_{sink} parameters of the myocardium model. The initialized parameters are maintained constant along all following coupling iterations unless otherwise stated.

In the coupling iterations, the resulting pressure of the coronary model at each terminal segment end $p^{\mathrm{T,i}}_k$ is used as input to the porous model, defining the source pressure in each tessellation territory Ω_i. The porous model is then solved for flow values for each perfusion territory, that are used as new inputs for the coronary model, $q^{\mathrm{T,i}}_{k+1}$. For iterations with $k > 0$, the coronary model solves for pressure with given flows while keeping the same geometry and boundary conditions. The coupling loop is illustrated in Fig. 1d (main paper). The

coupling convergence is established considering the terminal segment flow values between iteration k and iteration $k+1$:

$$\frac{\left|q_k^{\mathrm{T,i}} - q_{k+1}^{\mathrm{T,i}}\right|}{q_k^{\mathrm{T,i}}} < 1\% \text{ for all i} \tag{10}$$

In practice for a few cases, in particular the patient with significant obstructive disease, a relaxation scheme was necessary.

Note that in principle, each perfusion territory is assigned to one and only one segment. However in some cases, the scale of the segment outlets is much smaller than the resolution of the myocardial mesh. As a result, during the Voronoi tessellation computation, two segments can be assigned to the same perfusion territory, which can cause problems to the coupling algorithm. In order to avoid this, we locally refine the myocardial mesh and rerun the tessellation. If the issue persists as the segments are too close to each other and an excessively refined mesh resolution is required, we modify the vasculature by trimming the smallest terminal segment and recompute the tessellation.

4 Sensitivity to the synthetic vascular network

In this section, we assess the impact of the vascular network on the results by generating different synthetic vasculatures for the same patient (Patient 1) as in section 3.1 (main paper).

4.1 Varying number of terminal segments

We generated three distinct synthetic vasculatures with 3000, 6000 and 12000 terminal segments, respectively.

Vasculature analysis We evaluate the vascular depth reached in each vasculature using the Strahler order system[4]. Overall, increasing the number of terminal segments leads to a greater percentage of synthetic segments with lower Strahler orders (5-6) and a lower

percentage of segments with higher Strahler orders (7-9) (Fig. 4.1a). This implies that vasculatures with higher number of terminals achieved greater depth. In particular, close to 20% of synthetic segments in the 12000-terminals vasculature were extended down to Strahler order 5, while $< 5\%$ and $< 1\%$ of segments reached that order in the 3000 and 6000-terminal vasculatures, respectively.

Further investigating the diameter distribution of terminal segments reveals that both smaller diameters and greater terminal diameter uniformity is achieved with an increase in number of terminal vessels (Fig. 4.1b). Mean and standard deviation of terminal diameters are $195 \pm 48\,\mu m$, $155 \pm 38\,\mu m$ and $123 \pm 30\,\mu m$ for the 3000, 6000 and 12000 terminal vascular trees, respectively.

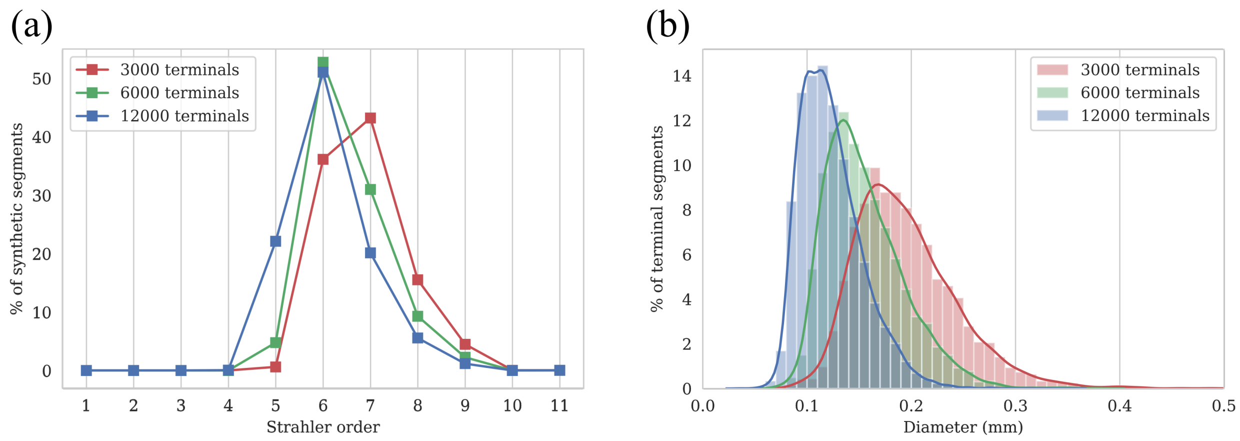

Figure 4.1: Geometrical analysis of vascular networks comprising 3000, 6000 and 12000 terminal segments. (a) Percentage of synthetic segments in each Strahler order. (b) Distribution of terminal segment diameters.

Hemodynamic results Blood flow in the three generated vasculatures is summarized in Table 3 (main paper). In terms of blood flow in the coronary arteries, increasing the number of terminals leads to a more homogeneous flow distribution at the outlet level for both coupled and coronary models, as suggested by the lower standard deviation of terminal flows (Table 3, main paper). This behaviour reflects the more homogeneous diameter distribution. Naturally, the mean terminal segment flow is also lower, as the same amount of total flow is

distributed in more segments.

In terms of blood flow in the myocardium, mean MBF remains almost identical regardless of the number of terminals and the model used. While the coronary model MBF values remain extremely heterogeneous, a slight reduction is observed when increasing the terminal segments (see reduced SD, Table 3, main paper). In contrast, the coupled model exhibits the same level of homogeneity regardless of vasculature, showing robustness to the variation in number of terminal segments.

The level of heterogeneity for both models is also illustrated in their respective perfusion maps: coronary model maps consist of significantly over- and under-perfused regions, while the coupled model ones are quite homogeneous. In terms of spatial distribution, although perfusion maps are not identical, the main perfusion features are present across vasculatures (Fig. 4.2).

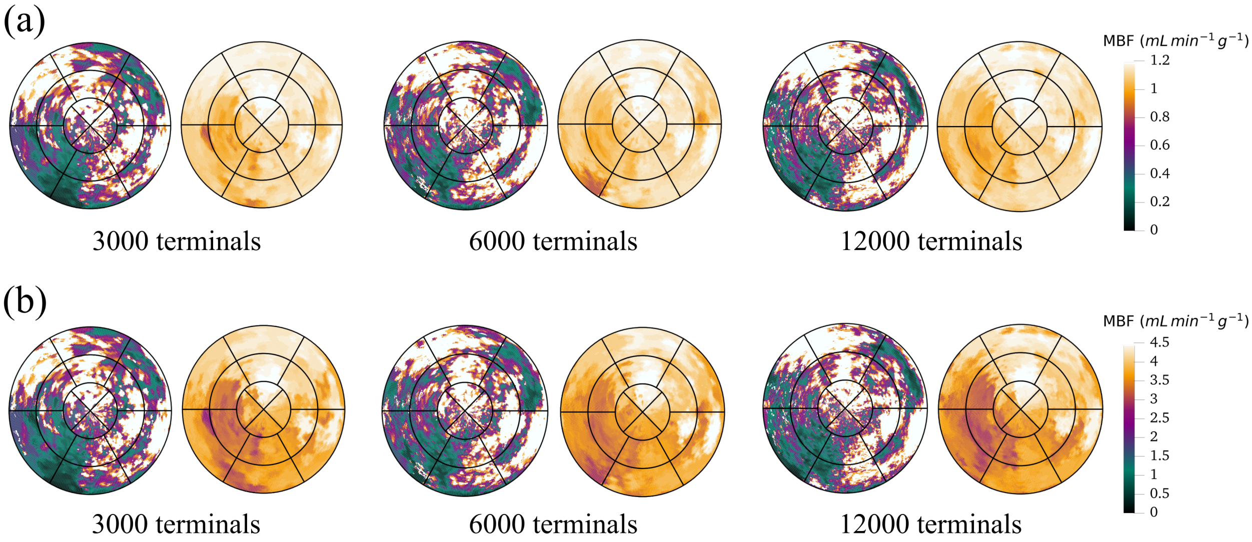

Figure 4.2: Simulated perfusion maps for vasculatures with varying number of terminal segments for (a) resting and (b) hyperemic conditions. For each vasculature: coronary model (left), coupled model (right). Note the upper limit of the color map matches the maximum MBF value of the coupled model across vasculatures. Coronary model MBF values in perfusion maps reach up to $77\,\mathrm{mL\,min^{-1}\,g^{-1}}$ and $243\,\mathrm{mL\,min^{-1}\,g^{-1}}$ in resting and hyperemic conditions, respectively, across vasculatures.

For all the above considered characteristics, varying the total number of terminal segments has a similar impact on the results in both resting and hyperemic conditions.

To conclude, the 12000-terminals vasculature demonstrates preferable morphological features (greater vascular depth, more uniform outlet diameter size) leading to a minor, albeit noticeable, improvement in the coronary model results. In order to explicitly model as many vessels as possible while maintaining a manageable additional computational cost, we consider 12000 terminal segments as the default choice for this paper.

4.2 Varying randomness of the vasculature

Here we generate 5 vasculatures comprising 12000 terminal segments. All synthetic trees start from the same roots on the segmented vessels, but grow differently based on the inherent randomness in the tree generation as described in[3]. Thus these 5 "seeds" lead to different but similar-looking synthetic networks.

MBF results for both models across different seeds are presented in Fig. 4.3. The choice of seed is only significant in the global flow heterogeneity of the coronary model (Fig. 4.3a, 4.3b). In fact, choosing a particular seed has a larger impact on the standard deviation of simulated MBF for the coronary model, compared to varying the number of terminal segments. The coupled model provides consistent results regardless of the seed, demonstrating robustness in the geometrical variability of the vasculature.

The associated perfusion maps display some regional differences, but their spatial distribution of flow has similar main characteristics (Fig. 4.3c). For example, regions from 7 to 9 o'clock consistently have lower MBF compared to the rest of the map. Results are similar for resting and hyperemic conditions.

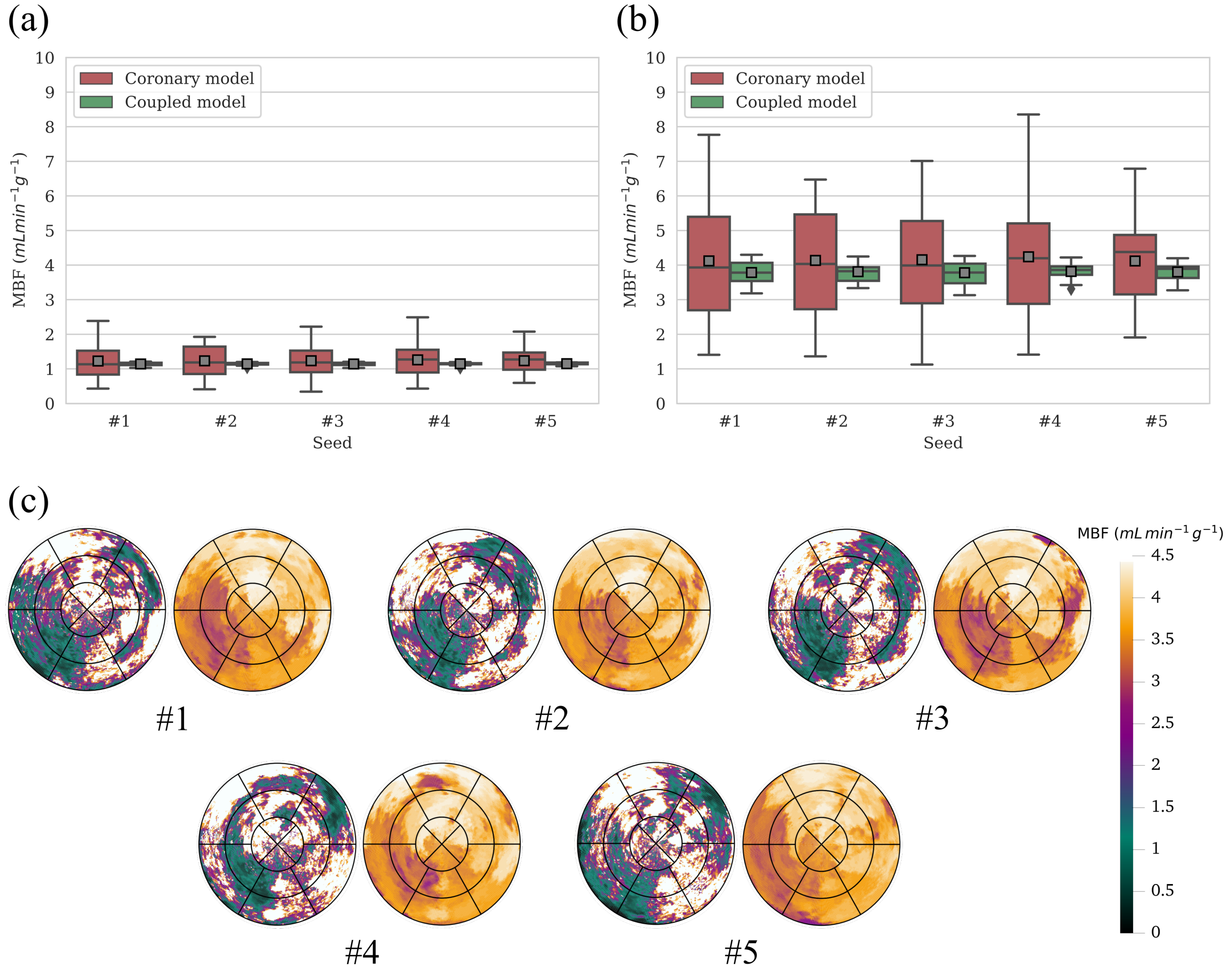

Figure 4.3: MBF$_{AHA}$ results for 5 random seeds for (a) resting and (b) hyperemic conditions. Mean MBF$_{AHA}$ values are depicted with grey squares. (c) Simulated perfusion maps for hyperemic conditions. For each seed: coronary model map (left), coupled model map (right). Note the upper limit of the color map matches the maximum MBF value of the coupled model across seeds. Coronary model MBF values in perfusion maps reach up to 225 $\mathrm{mL\,min^{-1}\,g^{-1}}$ across seeds.

5 Patient selection and characteristics

For this study, a total of 6 patients were selected to have high cCTA image quality and contain a wide distribution of heart dominance (3 right, 2 codominant, 1 left). They are grouped based on non-obstructive CAD (5 patients) or obstructive CAD (1 patient), with obstructive CAD defined as $> 30\%$ reduction in diameter in at least one location on the larger segmented vessels.

Table 1: Patient characteristics of 6 patients with suspected CAD who underwent cCTA and [^{15}O]H_2O Positron Emission Tomography (PET)[2] prior to invasive coronary angiography, which demonstrated non-obstructive CAD in 5 patients and obstructive CAD in 1 patient. ACE = angiotensin-converting enzyme; ARB = angiotensin receptor blocker.

	Patient 1	Patient 2	Patient 3	Patient 4	Patient 5	Patient with obstructive CAD
Age	54	59	59	56	46	53
Sex	female	female	male	male	female	male
Body mass index (kg m^{-2})	22.95	28.08	27.90	20.38	29.32	25.93
Heart dominance	right	right	codominant	codominant	left	right
Comorbidities						
Current smoker	No	No	No	Yes	No	No
Smoking history	No	No	No	Yes	No	Yes
Diabetes (type I)	No	No	No	No	No	No
Diabetes (type II)	No	No	No	No	No	No
Hypertension	Yes	No	No	No	No	No
Hypercholesterolemia	No	Yes	No	No	No	Yes
Family history of CAD	Yes	No	No	Yes	Yes	No
Medications						
Acetylic acid	Yes	Yes	Yes	Yes	No	Yes
Statin	Yes	Yes	Yes	Yes	No	Yes
ACE inhibitors	No	No	Yes	No	No	Yes
ARBs	Yes	No	No	No	No	No
Long acting nitrates	Yes	No	No	No	No	No
Beta blockers	Yes	No	No	Yes	No	Yes
Calcium channel blockers	Yes	No	No	No	No	Yes
Symptoms	atypical angina	aspecific chest pain	atypical angina	typical angina	aspecific chest pain	typical angina

6 Additional illustrations

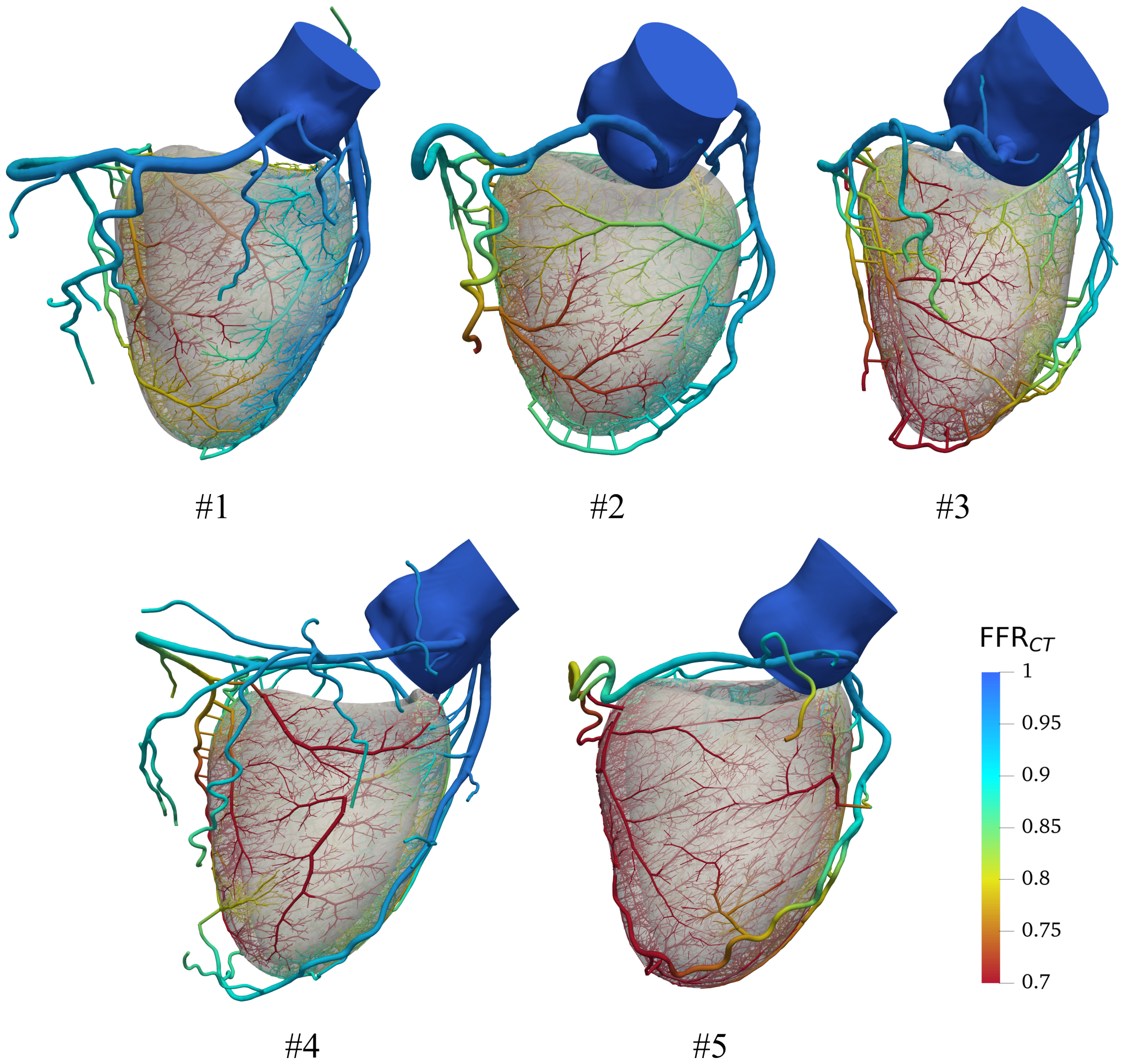

Figure 6.1: FFR$_{\mathrm{CT}}$ analysis along the segmented and synthetic vasculature for 5 patients with non-obstructive CAD (hyperemic conditions).

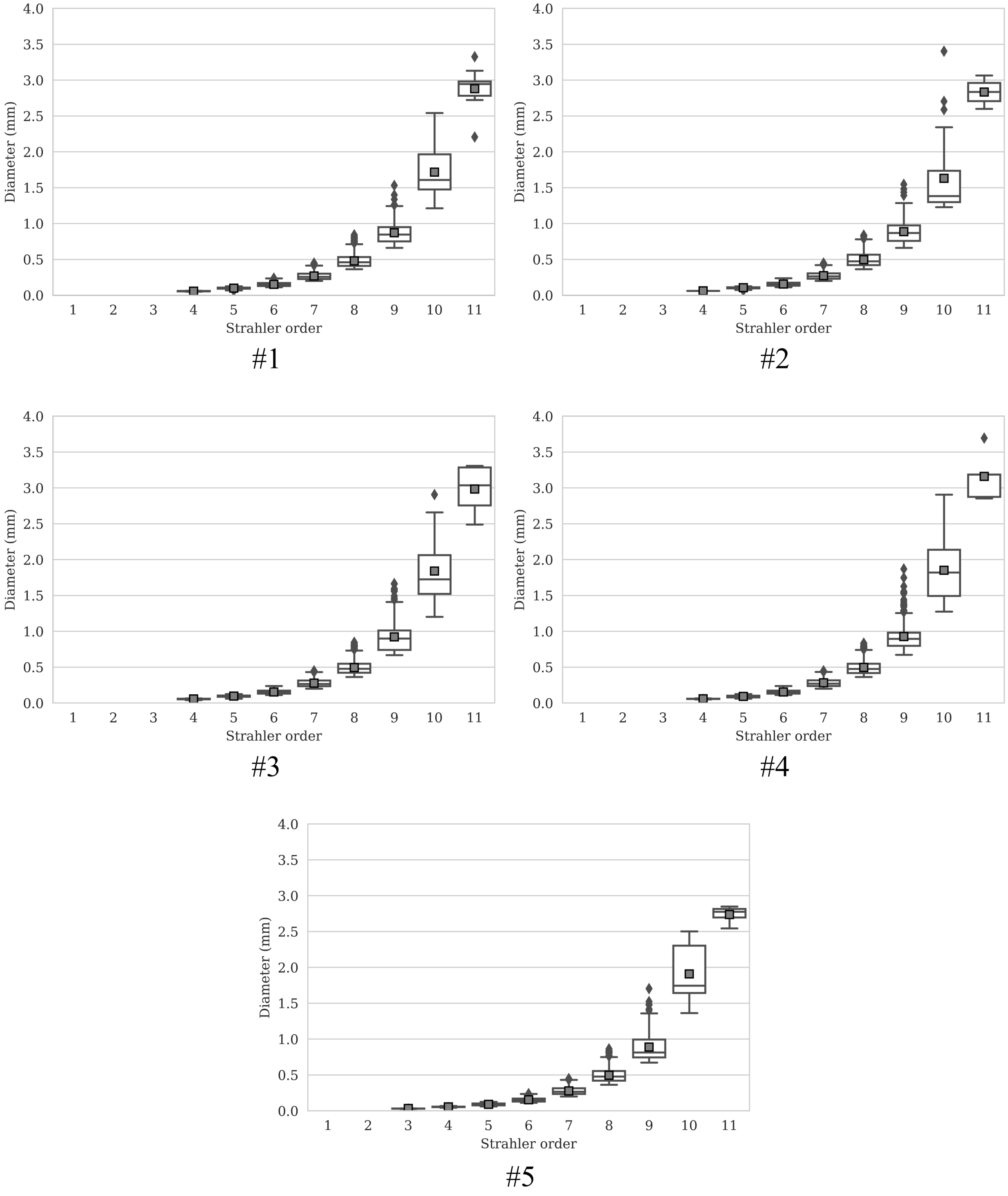

Figure 6.2: Diameter distribution of segmented vessels (orders 9-11) and undilated synthetic segments (orders 3-9) within each Strahler order for five patients with non-obstructive CAD. Vasculatures comprise 12000 terminal segments.

References

1. Danad, I., P. G. Raijmakers, R. S. Driessen, J. Leipsic, R. Raju, C. Naoum, J. Knuuti, M. Mäki, R. S. Underwood, J. K. Min, K. Elmore, W. J. Stuijfzand, N. van Royen, I. I. Tulevski, A. G. Somsen, M. C. Huisman, A. A. van Lingen, M. W. Heymans, P. M. van de Ven, C. van Kuijk, A. A. Lammertsma, A. C. van Rossum, and P. Knaapen. Comparison of coronary CT angiography, SPECT, PET, and hybrid imaging for diagnosis of ischemic heart disease determined by fractional flow reserve. JAMA Cardiology 2:1100–1107, 2017.

2. Danad, I., V. Uusitalo, T. Kero, A. Saraste, P. G. Raijmakers, A. A. Lammertsma, M. W. Heymans, S. A. Kajander, M. Pietilä, S. James, J. Sörensen, P. Knaapen, and J. Knuuti. Quantitative assessment of myocardial perfusion in the detection of significant coronary artery disease: Cutoff values and diagnostic accuracy of quantitative [15O]H2O pet imaging. Journal of the American College of Cardiology 64:1464 – 1475, 2014.

3. Jaquet, C., L. Najman, H. Talbot, L. Grady, M. Schaap, B. Spain, H. J. Kim, I. Vignon-Clementel, and C. A. Taylor. Generation of patient-specific cardiac vascular networks: a hybrid image-based and synthetic geometric model. IEEE Transactions on Biomedical Engineering 66:946–955, 2019.

4. Kassab, G. S., C. A. Rider, N. J. Tang, and Y. C. Fung. Morphometry of pig coronary arterial trees. American Journal of Physiology-Heart and Circulatory Physiology 265:H350–H365, 1993.

5. Wilson, R. F., K. Wyche, B. V. Christensen, S. Zimmer, and D. D. Laxson. Effects of adenosine on human coronary arterial circulation. Circulation 82:1595–1606, 1990.
